# Curcumin inhibits ferroptosis through dessuccinylation of SIRT5-associated ACSL4 protein, and plays a chondroprotective role in osteoarthritis

**DOI:** 10.1371/journal.pone.0328139

**Published:** 2025-08-18

**Authors:** Yong Xu, Yongxia Li, Lei Liu, Qingling Jing, Xiaojian Ye

**Affiliations:** 1 Department of Orthopedics, Tongren Hospital Affiliated to Shanghai Jiao Tong University School of Medicine, Shanghai, China; 2 Department of Anesthesiology, Affiliated Hospital of Qinghai University, Qinghai, China; 3 Department of Orthopedics Surgery, Affiliated Hospital of Qinghai University, Qinghai, China; The Affiliated Changzhou No 2 People's Hospital of Nanjing Medical University, CHINA

## Abstract

**Background:**

Ferroptosis of chondrocytes plays a crucial role in the progression of osteoarthritis (OA). This study aimed to explore the role of curcumin (Cur) in interfering with chondrocyte ferroptosis in OA.

**Methods:**

Rat chondrocytes were treated with 10 ng/mL interleukin-1β (IL-1β) for 48 hours to mimic the OA microenvironment. The protective effects of Cur were evaluated in vitro by assessing cell viability and ferroptosis. Molecular docking was performed to validate the structural interaction between Cur and the SIRT5 protein. Co-immunoprecipitation (CO-IP) confirmed the binding relationship between SIRT5 and ACSL4. Additionally, the efficacy of Cur in alleviating OA progression was assessed in an in vivo OA rat model.

**Results:**

Cur treatment significantly attenuated IL-1β-induced chondrocyte injury by enhancing cell viability and inhibiting ferroptosis. Cur also markedly reduced global protein lysine succinylation levels. IL-1β suppressed SIRT5 expression, while Cur treatment upregulated SIRT5 expression. The molecular structure of Cur exhibits strong complementarity with the SIRT5 protein, forming a stable complex with high binding affinity. Inhibition of SIRT5 attenuated the protective effects of Cur on chondrocytes and increased ACSL4 succinylation levels. SIRT5 physically interacted with ACSL4, and SIRT5-mediated desuccinylation of ACSL4 repressed its function, thereby mitigating ferroptosis. Cur alleviates OA progression *in vivo* by inhibiting cartilage destruction, bone erosion, and chondrocyte injury, and by smoothing subchondral bone surfaces.

**Conclusion:**

Cur protects chondrocytes in vitro by inhibiting ferroptosis and suppresses cartilage degeneration and bone erosion in vivo, demonstrating a chondroprotective role in OA. These effects are mediated through SIRT5-dependent desuccinylation of ACSL4, which regulates ferroptosis pathways.

## Introduction

Osteoarthritis (OA) is a prevalent chronic degenerative disorder representing a significant health burden for the elderly population [1]. Although the pathogenesis of OA is multifactorial, the progressive destruction and degradation of the cartilage extracellular matrix, triggered by factors such as inflammation and mechanical trauma, are considered central to disease onset and progression [2]. As the sole cellular component of cartilage, chondrocytes play a central role in maintaining cartilage integrity and regulating matrix homeostasis [3]. The death of chondrocytes via various programmed cell death pathways disrupts the equilibrium between anabolic synthesis and catabolic degradation of critical extracellular matrix components, including collagen and glycosaminoglycans. This imbalance ultimately leads to cartilage matrix depletion, structural disintegration of articular cartilage, and the clinical manifestations of OA [4–6].

Ferroptosis is an iron-dependent form of regulated cell death characterized by lipid peroxidation. Accumulating evidence indicates that elevated intracellular iron levels exacerbate the progression of OA in animal models [7–9]. Furthermore, ferroptosis has been identified in human OA tissues, suggesting its involvement in disease pathogenesis [10]. Targeting ferroptosis by modulating the expression of ferroptosis-related genes represents a promising therapeutic strategy to alleviate OA [10,11]. Curcumin (Cur) is a bioactive polyphenolic compound derived from turmeric (Curcuma longa), renowned for its multifaceted pharmacological properties, including anti-tumor, anti-inflammatory, antioxidant, anti-fibrotic, and cardioprotective effects [12–14]. Emerging studies highlight its potential in OA management [15–17]. Notably, Cur has been shown to upregulate nuclear factor erythroid 2-related factor 2 (Nrf2), thereby suppressing chondrocyte ferroptosis and mitigating cartilage degeneration [18]. Given these findings, this study aims to elucidate the molecular mechanisms underlying Cur’s protective effects against OA by targeting ferroptosis in chondrocytes.

Post-translational modifications (PTMs) of ferroptosis-related proteins are key regulatory mechanisms governing ferroptosis. For instance, the deubiquitinating enzyme OTUB1 directly interacts with SLC7A11 to inhibit its ubiquitination, thereby stabilizing SLC7A11 and suppressing ferroptosis in cancer cells [19]. GPX4, a central regulator of ferroptosis, undergoes diverse PTMs, including ubiquitination, succinylation, phosphorylation, and glycosylation, which modulate its stability and enzymatic activity [20]. Succinylation is a PTM involving the covalent addition of succinyl groups to lysine residues of target proteins. This modification is widespread in organisms and participates in diverse biological processes, such as lipid metabolism regulation, epigenetic control, and signaling dysregulation [21]. Given that lipid peroxidation accumulation is a cardinal driver of ferroptosis, succinylation—a metabolism-linked PTM—may play a role in its regulation. For example, studies have shown that the TP53-induced glycolysis and apoptosis regulator (TIGAR) suppresses neuronal ferroptosis by inhibiting succinate dehydrogenase (SDH) activity through acetylation and succinylation-mediated modifications [22]. Collectively, these findings suggest that succinylation of ferroptosis-related proteins may represent a critical regulatory axis. Therefore, we hypothesize that Cur could mitigate chondrocyte ferroptosis and alleviate OA progression by modulating the succinylation status of key ferroptosis-associated proteins.

In this study, we investigated the effects of Cur on suppressing chondrocyte ferroptosis and alleviating OA progression in both in vitro and in vivo models. Furthermore, we explored whether Cur treatment could modulate global succinylation levels and the succinylation status of ferroptosis-related proteins. The regulatory mechanism of Cur was further validated through molecular docking analysis. This study aims to provide a theoretical foundation for Cur’s therapeutic application in OA and to identify potential molecular targets underlying its protective effects against cartilage degeneration.

## Methods and materials

### Cell culture and treatment

Rat chondrocytes (CP-R092, Procell, Wuhan, Hubei, China) were cultured in rat chondrocyte complete medium (CM-R087, Procell, Wuhan, Hubei, China) supplemented with 15% fetal bovine serum (FBS, Gibco) at 37°C in a humidified incubator with 5% CO₂. An *in vitro* inflammatory model mimicking OA was established by exposing chondrocytes to 10 ng/mL rat interleukin-1 beta (IL-1β, 501-RL, R&D Systems, Minneapolis, MN, USA) for 48 hours [23]. For Cur treatment, cells were pretreated with 10 μM Cur (Cat. #C1386, Sigma-Aldrich, St. Louis, MO, USA) dissolved in phosphate-buffered saline (PBS) for 12 hours prior to IL-1β stimulation [24,25]. Adenoviruses for overexpression or knockdown of SIRT5 and ACSL4, along with corresponding negative controls, were purchased from HedgehogBio (Shanghai, China). Chondrocytes were infected with adenoviruses following the manufacturer’s protocol. After 48 hours of transfection, cells were treated with Cur and/or IL-1β as indicated.

### Cell viability and ferroptosis evaluation

The protective effects of Cur on chondrocytes were assessed by evaluating cell viability and ferroptosis. Cell viability was determined using the Cell Counting Kit-8 (CCK-8) assay. Chondrocytes were seeded into 96-well plates at a density of 2 × 10⁵ cells/mL. Following transfection, cells treated with IL-1β in the presence or absence of Cur were incubated with 10 μL of CCK-8 reagent (C0042, Beyotime, Shanghai, China) at 37°C for 2 hours. Absorbance values were measured at 450 nm using a microplate reader (Synergy H1, BioTek, Winooski, VT, USA). Ferroptosis was assessed by evaluating cell death, reactive oxygen species (ROS), malondialdehyde (MDA), glutathione (GSH), and ferrous iron (Fe^2+^) levels. Cell death was quantified via DAPI/PI double staining. After centrifugation, cells were resuspended in 1 mL of cell staining buffer, followed by incubation with 5 μL DAPI and 5 μL PI staining solutions (Beyotime) at 4°C for 30 minutes. Stained cells were visualized using a fluorescence microscope (TE2000, Nikon, Japan). Intracellular ROS production was measured using the DCFH-DA fluorescent probe and a ROS assay kit (S0033, Beyotime). Fe^2+^, GSH, and MDA concentrations were determined using iron assay kits (ab83366, Abcam, Cambridge, MA, USA), GSH assay kits (ab65322, Abcam), and MDA enzyme-linked immunosorbent assay (ELISA) kits (ab287797, Abcam), respectively, following the manufacturers’ protocols.

### Western blotting, immunoprecipitation (IP) and co-immunoprecipitation (CO-IP) method

Total protein of chondrocytes were collected with the help of RIPA reagent (Thermo Fisher Scientific, California, USA), and concentration were determined by the BCA Protein Assay Kits (Beyotime). Protein bands were obtained using the western blotting method. In brief, aprotein (20 μg) was separated by 10% SDS-PAGE and electrotransferred to PVDF membrane (Merck Millipore; Billerica, MA, USA). The membranes were blocked with 5% skim milk for an hour and incubated with primary antibodies at 4°C overnight followed by horseradish peroxidase-conjugated secondary antibodies for an hour. The total grey scale of each strip was quantified by ImageJ software with the values normalized based on the house‐keeping protein GAPDH. To access the succinylation of lysine (K) of proteins, Protein G Agarose beads (20398, Thermo Fisher) were added to the mixture of cell lysate and antibodies at room temperature for an hour. The resulting beads were boiled at 95 °C for 5 min in 35 µL SDS sample buffer. Both the input and immunoprecipitated samples were subjected to western blotting analysis. To examine the exogenous interaction between ACSL4 and SIRT5, cells were co-transfected with Flag-tagged SIRT5 (Flag-SIRT5) and HA-tagged ACSL4 (HA-ACSL4). Briefly, the coding sequences of rat SIRT5 and ACSL4 were amplified by PCR from cDNA templates using gene-specific primers. The PCR products were then cloned into mammalian expression vectors containing the Flag or HA epitope tag sequences at the N-terminus. The constructs were verified by DNA sequencing to ensure the correct insertion of the target genes and epitope tags. The resulting plasmids, Flag-SIRT5 and HA-ACSL4, were purified using a plasmid extraction kit and used for transfection experiments. After 48 h of transfection, cells were harvested and lysed in ice-cold lysis buffer (50 mM Tris-HCl, pH 7.4, 150 mM NaCl, 1% NP-40, 1 mM EDTA, and protease inhibitor cocktail) for 30 min on ice. The cell lysates were centrifuged at 12,000 × g for 15 min at 4°C to remove debris. The supernatant was then incubated with anti-Flag or anti-HA antibody-conjugated agarose beads overnight at 4°C with gentle rotation. The beads were washed three times with lysis buffer to remove non-specific binding. The immunoprecipitated proteins were eluted by boiling in 2 × SDS-PAGE loading buffer and subjected to western blotting analysis. As for protein stability, cycloheximide (CHX, 50 mg/mL; Selleck Chemicals; Houston, TX, USA) was added [26], and the samples were collected at 0, 8, 16, and 24 h, and the cell proteins were extracted. The expression of ACSL4 was detected by western blotting analysis. Information of all antibodies used in this study are listed as follows: rabbit anti-SLC7A11 monoclonal antibody (ab307601, 1/1000), rabbit anti-GPX4 monoclonal antibody (ab125066, 1/1000), rabbit anti-ACSL4 monoclonal antibody (ab155282, 1/10000), rabbit anti-KAT3B monoclonal antibody (ab259330, 1/1000), rabbit anti-CPT1A monoclonal antibody (ab234111, 1/1000), rabbit anti-HAT1 monoclonal antibody (ab194296, 1/1000), rabbit anti-SIRT5 monoclonal antibody (ab259967, 1/1000), rabbit anti-SIRT7 monoclonal antibody (ab259968, 1/1000), rabbit anti-SLC7A11 monoclonal antibody (ab307601, 1/1000), rabbit anti-FTH1 monoclonal antibody (ab183781, 1/1000), anti-COL2A1 monoclonal antibody (ab307674, 1/1000), anti-MMP13 antibody (ab39012, 1/3000), and rabbit anti-GAPDH monoclonal antibody (ab181602, 1/10000) were obtained from Abcam. Rabbit anti-KAT2A monoclonal antibody (ABE1418, 1/500), rabbit anti-TFR1 monoclonal antibody (SAB4200398, 1/400), normal rabbit IgG (NI01) was obtained from Merck Millipore (Shanghai, China). Rabbit anti-succinyllysine polyclonal antibody (PTM-401, 1/500) was purchased from PTM Biolabs (Hangzhou, Zhejiang, China).

### Dual-luciferase reporter assay

The promoter region of the SIRT5 gene was cloned upstream of the firefly luciferase gene in the pGL3-Basic vector (Promega, Madison, WI, USA) to construct a dual-luciferase reporter system. The pRL-TK plasmid (Promega), encoding Renilla luciferase, was used as an internal control to normalize transfection efficiency. Cells were seeded at a density of 1 × 10⁵ cells/well in 24-well plates and allowed to adhere overnight. Transfections were performed using Lipofectamine 3000 (Thermo Fisher Scientific, Waltham, MA, USA) following the manufacturer’s protocol. Each well received 200 ng of the firefly luciferase reporter plasmid and 20 ng of the Renilla luciferase control plasmid. After 24 hours of transfection, cells were lysed using Passive Lysis Buffer (PLB, Promega). Luciferase activities were sequentially measured using the Dual-Luciferase Reporter Assay System (Promega) on a luminometer (Berthold Technologies, Bad Wildbad, Germany). Firefly luciferase activity was normalized to Renilla luciferase activity to account for variations in transfection efficiency. All experiments were performed in triplicate.

### Molecular docking

The molecular structure of Cur compounds (458-37-7) was from the PubChem database (https://pubchem.ncbi.nlm.nih.gov/). Chem3D was used for format conversion and energy minimization, and then all structures were imported into Schrodinger software to establish a database, and energy minimization was optimized through hydrogenation structure, and saved as a ligand molecular database for molecular docking. SIRT5 (PDB ID:7X3P) protein structure was from the RCSB database (https://www.rcsb.org/). The protein structure was treated in the Maestro11.9 platform, and the protein was treated with Schrodinger’s Protein Preparation Wizard, which removed the crystal water, supplemented the missing hydrogen atoms, repaired the missing bond information, repaired the missing peptide segment, and finally minimized the energy of the protein and optimized the geometric structure. Molecular docking is the Protein processing done by the Glide module in the Schrödinger Maestro software using the Protein Preparation Wizard module.

### Prediction of potential succinylation sites

The Generic and Species-specific Succiniylation Sites Prediction Server (GPSuc, http://kurata14.bio.kyutech.ac.jp/GPSuc/index.php) was used to predict the potential succinylation sites of rat ACSL4 protein [27]. Mutation of ACSL4 mRNA was achieved with the help of Quickchange Site-Directed Mutagenesis Kit (Agilent Technologies; Santa Clara, CA, USA).

### Animal grouping and treatment

Eight-week-old male Sprague-Dawley (SD) rats weighing between 220–250 g were obtained from Charles river Biotechnology Co., LTD (Zhejiang, China). The rats were randomly divided into sham group and OA group, and OA + Cur treatment group (n = 6, each group). On day 0, rats were anesthetized with isoflurane inhalation at an initial induction concentration of 4−5% and a maintenance concentration of 2-2.5%. The rats were completely anesthetized and showed no pain response. After successful anesthesia, the rats were placed on the operating table, supine position was taken, and the knee joint of the lower limb was taken as the surgical area. The surgical area was routinely skinned, and the skin of the incision area was cleaned with 75% alcohol for disinfection. The OA model of knee was established by anterior cruciate ligament transection (ACLT). In sham group, only the articular capsule was cut and the anterior cruciate ligament was not cut. After the rats were awake, they were fed back into the cage and allowed to move freely without fixing the lower limb, and the respiratory tract of all the rats was ensured. For the first three days after surgery, penicillin was used. According to the clinical characteristics that fatigue activities are prone to OA in advance, after the successful modeling, the rats were forced to exercise 20 mim every day for 3 consecutive weeks. After 2 weeks of surgery, rats of each group underwent knee joint cavity injection for 8 weeks. Rats in sham and OA group were injected with 100 μL PBS twice a week, rats in OA + Cur group were injected with 100 μL Cur (100 μg/mL) twice a week [28]. The animal experiments in this study were performed following the NIH Guide for the Care and Use of Laboratory Animals and approved by the Ethics Committee of Shanghai Tong Ren Hospital (A2024-011-01), and conducted in accordance with the ethical guidelines for animal welfare in China. All rats were euthanized at week 10 by placing them in a sealed euthanasia box, gradually introducing carbon dioxide gas to make them unconscious, and ultimately euthanizing them.

### Evaluation of OA progress

The knee-joints of rats were fixed with 4% paraformaldehyde for 24 h and then scanned by a Micro-CT system (skyscan1275, Brucker, Belgium). An X-ray source at 60 kV was set, the knee-joints were then scanned. Several bone-related parameters were analyzed, including bone volume/total volume (BV/TV, %) and bone mineral density (BMD, g/dm^3^).

### Specimen preparation and histological evaluation

The knee joint were taken, and fixed in 10% formalin at 4°C for 24 h. After complete decalcification using EDTA-Na_2_ solution, the tissues were dehydrated in paraffin. Paraffin sections (5 μm) were stained with hematoxylin and eosin (H&E) and Safranin O/fast green. The Osteoarthritis Research Society International (OARSI) score system was used to assess the osteoarthritis score of the joint tissue.

### RNA extraction and quantitative real-time PCR analysis

The TRIzol reagent (Invitrogen; Carlsbad, CA, USA) was used to to extract the total RNA from cells or tissues. The integrity of RNA was detected by electrophoresis with 1% agarogel. The optical density D(λ) at 260 nm and 280 nm was determined by a microplate reader (800TS; Bio-Tek; Winooski, Vermont, USA), and D(λ)260/D(λ)280 was controlled in the range of 1.8 ~ 2.0. Reverse transcription kit (TaKaRa; Tokyo, Japan) was used to reverse RNA into cDNA. The specific operation was carried out according to the instructions. The expressions of SIRT5, ACSL4, COL2A1 and MMP13 mRNA in knee tissues were detected by qR-TPCR. Reaction system: 2 μL cDNA, 1 μL upstream and downstream primers, 12.5 μL TB Green Premix Ex Taq^TM^Ⅱ and 8.5 μL sterilized water. Reaction conditions: predenaturation at 95 °C for 2 min; 40 cycles were set up at 95 °C for 15 s and 60 °C for 30 s. The relative expression of target genes was calculated by 2^-ΔΔCt^ method with GAPDH as the internal reference. The primers sequence is shown below: rat SIRT5: Forward 5′-CCTTTTGCAGCCTGCCC-3′, Reverse 5′-TGCGTTCGCAAAACACTTCC-3′; rat ACSL4: Forward 5′-CCTGAGGGGCTTGAAATTCAC-3′, Reverse 5′-GTTGGTCTACTTGGAGGAACG-3′; rat COL2A1: Forward 5′-GGGTCACAGAGGTTACCCAG-3′, Reverse 5′-ACCAGGGGAACCACTCTCAC-3′; rat MMP13: Forward 5′-TGTTTGCAGAGCACTACTTGAA-3′, Reverse 5′-CAGTCACCTCTAAGCCAAAGAAA-3′; rat GAPDH: Forward 5′-AATGGATTTGGACGCATTGGT-3′, Reverse 5′-TTTGCACTGGTACGTGTTGAT-3′.

### Statistical analysis

All experiments were conducted independently at least three times. Data analysis was performed using GraphPad Prism 9.0 software, and results are presented as the mean ± standard deviation (SD). Prior to statistical analysis, data were checked for normality using the Shapiro-Wilk test. For multi-group comparisons, one-way analysis of variance (ANOVA) was employed. If the ANOVA indicated significant differences among groups, post-hoc pairwise comparisons were conducted using Student’s t-test with a Bonferroni correction to adjust for multiple comparisons. A p-value threshold of < 0.05 was used to determine statistical significance.

## Results

### Cur treatment protects chondrocytes from IL-1β induced injury by promoting cell viability and inhibiting cell ferroptosis

**Fig 1:**
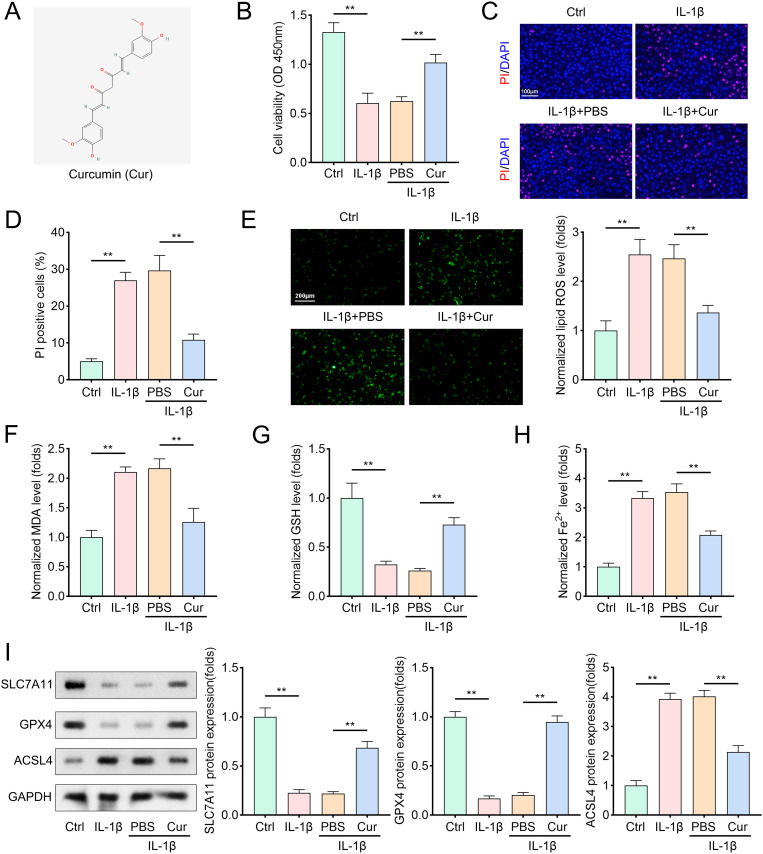
Cur treatment protects chondrocytes from IL-1β induced injury by promoting cell viability and inhibiting cell ferroptosis. (A) The chemical structural formula of Cur. (B) Cell viability of chondrocytes obtained by CCK-8 method. Cells were treated with 10 μM Cur for 12 hours hours prior to IL-1β stimulation (10 ng/mL, 48 hours) and then incubated with CCK-8 solution. The absorbance at 450 nm was measured using a microplate reader to quantify viable cells, n = 3. (C–D) DAPI/PI staining method was applied to evaluate cell death of condrocytes. After treatment, cells were fixed, permeabilized, and stained with DAPI and PI. Images were captured using fluorescence microscopy, n = 3. (red: PI-labeled dead cells; blue: DAPI-labeled nuclei). (E–H) Cell ferroptosis of chondrocytes was accessed by evaluating ROS, MDA, GSH, and Fe^2+^ levels. Intracellular ROS levels were measured using DCFH-DA fluorescence. MDA levels were determined through a thiobarbituric acid assay. GSH levels were measured using an enzymatic recycling method. Fe^2+^ levels were quantified with a ferrozine-based assay, n = 3. (I) Representative protein bands and quantitative analysis of GPX4 and ACSL4 levels in chondrocytes. Protein samples were extracted, separated by SDS-PAGE, transferred to PVDF membranes, and incubated with specific antibodies against GPX4 and ACSL4. Band intensities were analyzed using ImageJ software, n = 3. Data were presented as mean ± SD. Comparisons between groups were performed using one-way ANOVA followed by Tukey’s post-hoc test. **p < 0.01.

Cur, a polyphenolic compound derived from the rhizomes of Curcuma longa (chemical formula: C21H20O6, Fig 1A), was evaluated for its effects on chondrocytes. CCK-8 assay results demonstrated that IL-1β treatment significantly reduced chondrocyte viability and increased the number of propidium iodide (PI)-stained cells compared to the control (Ctrl) group (Fig 1B–1D). Cur treatment mitigated IL-1β-induced cytotoxicity by enhancing cell viability and reducing PI-stained cell counts. Additionally, IL-1β-induced ferroptosis in chondrocytes was characterized by elevated levels of lipid ROS, MDA, and Fe2 + , alongside decreased GSH levels (Fig 1E–1H). Cur treatment significantly reversed these effects: it reduced lipid ROS, MDA, and Fe2 + levels while increasing GSH levels compared to IL-1β-treated cells. Western blot analysis further revealed that IL-1β downregulated the expression of SLC7A11 and GPX4, while upregulating ACSL4. Cur treatment antagonized these changes, restoring ferroptosis-related protein levels to near-control values (Fig 1I). Collectively, these findings demonstrate that Cur exerts protective effects against IL-1β-induced chondrocyte injury through inhibition of ferroptosis.

### The molecular structure of Cur has a good binding effect on the target protein SIRT5

**Fig 2:**
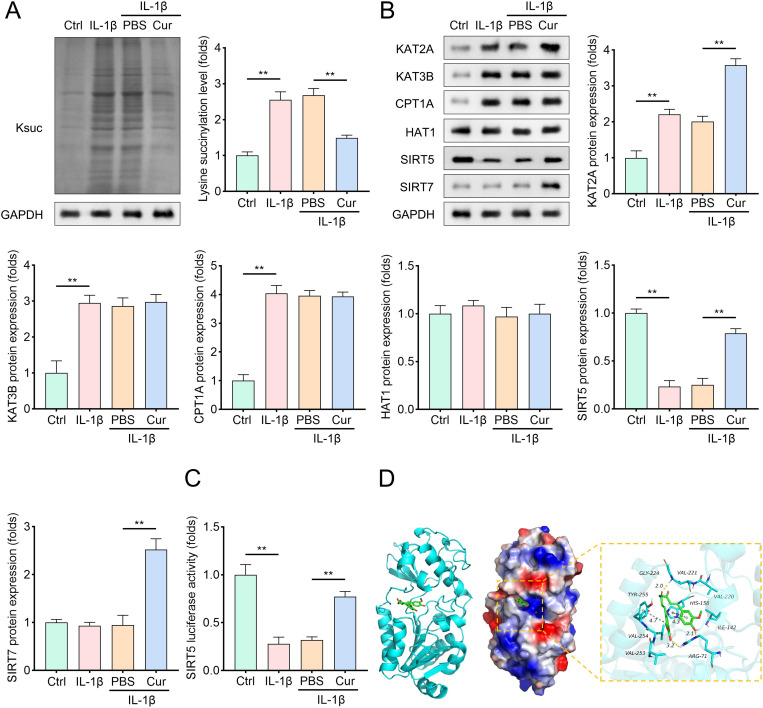
Cur reduces global protein succinylation and upregulates SIRT5 expression, with molecular docking suggesting a direct interaction between Cur and SIRT5. (A) The representative protein images and quantitative analysis of total succinylation of lysine of proteins in chondrocytes. Cells were lysed, protein samples were prepared and subjected to Western blot analysis using anti-succinyllysine antibodies, n = 3. (B) The representative protein images and quantitative analysis of protein levels of four succinylase (KAT2A, KAT3B, CPT1A, and HAT1) and two desuccinylase (SIRT5 and SIRT7) in chondrocytes of each group. Protein levels were determined using Western blot assay with specific antibodies, n = 3. (C) The enzyme activity of SIRT5 in chondrocytes before and after Cur treatment. SIRT5 activity was measured using a commercially available SIRT5 activity assay kit according to the manufacturer’s instructions, n = 3. (D) The molecular docking results suggested that structure of Cur compound is well matched with SIRT5 target protein. Docking studies were performed using molecular docking software to predict the binding mode and affinity between Cur and SIRT5. Data were presented as mean ± SD. Comparisons between groups were performed using one-way ANOVA followed by Tukey’s post-hoc test. **p < 0.01.

Previous studies have highlighted the role of protein succinylation in ferroptosis and other pathophysiological processes [22,29]. We hypothesized that Cur might regulate chondrocyte ferroptosis via succinylation modification. As shown in Fig 2A, IL-1β treatment significantly increased global lysine succinylation levels in chondrocytes, whereas Cur treatment markedly reduced this effect. To identify the molecular mechanism, we analyzed the expression of four succinyltransferases (KAT2A, KAT3B, CPT1A, and HAT1) and two desuccinylases (SIRT5 and SIRT7) in chondrocytes from each group. Among these enzymes, SIRT5 expression was suppressed by IL-1β but restored by Cur treatment (Fig 2B). Further, IL-1β decreased the luciferase activity of SIRT5 in chondrocytes while Cur treatment enhanced its activity (Fig 2C), suggesting a direct interaction between Cur and SIRT5. Molecular docking analysis revealed that the Cur structure exhibited excellent complementarity with the SIRT5 protein, forming a stable complex with strong binding affinity (Fig 2D). These findings indicate that Cur’s anti-ferroptotic effects may arise from its ability to modulate SIRT5-mediated protein succinylation.

### Inhibition of SIRT5 weakens the protecting effects of Cur on chondrocytes

**Fig 3:**
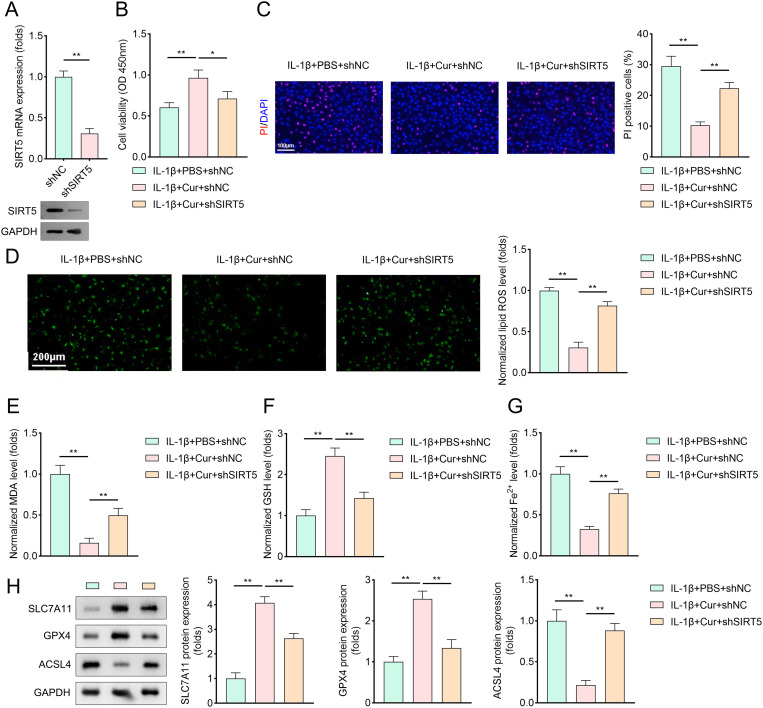
Inhibition of SIRT5 weakens the protecting effects of Cur on chondrocytes. (A) PCR and western blotting analysis were performed to evaluate the levels of SIRT5 in chondrocytes transfected with shNC or shSIRT5. Total RNA was extracted, reverse-transcribed into cDNA, and amplified using specific primers for SIRT5. Protein samples were also collected and subjected to Western blot analysis with SIRT5-specific antibodies, n = 3. (B) Cell viability of chondrocytes transfected with shNC or shSIRT5 were obtained by CCK-8 method after IL-1β treatment, n = 3. (C) DAPI/PI staining method was applied to evaluate cell death of IL-1β treated chondrocytes (transfected with shNC or shSIRT5), n = 3. (red: PI-labeled dead cells; blue: DAPI-labeled nuclei). (D–G) Cell ferroptosis of IL-1β treated chondrocytes (transfected with shNC or shSIRT5)was accessed by evaluating ROS, MDA, GSH, and Fe^2+^ levels, n = 3. (H) Representative protein bands and quantitative analysis of GPX4 and ACSL4 levels in IL-1β treated chondrocytes (transfected with shNC or shSIRT5), n = 3. Data were presented as mean ± SD. Comparisons between groups were performed using Student’s t-test or one-way ANOVA followed by Tukey’s post-hoc test. *p < 0.05, **p < 0.01.

To validate the role of SIRT5 in Cur’s protective effects, we first downregulated SIRT5 expression in chondrocytes using shSIRT5, the silencing efficiency was confirmed by qRT-PCR and western blotting (Fig 3A). Subsequent experiments revealed that SIRT5 inhibition significantly reversed the Cur-induced increases in cell viability and reductions in propidium iodide (PI)-stained cell counts compared to Cur-treated cells (Fig 3B–3D). Furthermore, Cur treatment reduced levels of lipid reactive oxygen species (ROS), malondialdehyde (MDA), Fe2 + , and ACSL4, while increasing SLC7A11 and GPX4 expression. However, SIRT5 inhibition notably attenuated these beneficial effects of Cur on ferroptosis-related parameters (Fig 3E–3H).

### Repression of ACSL4 function by SIRT5-mediated desuccinylation in response to cell ferroptosis of chondrocytes

**Fig 4:**
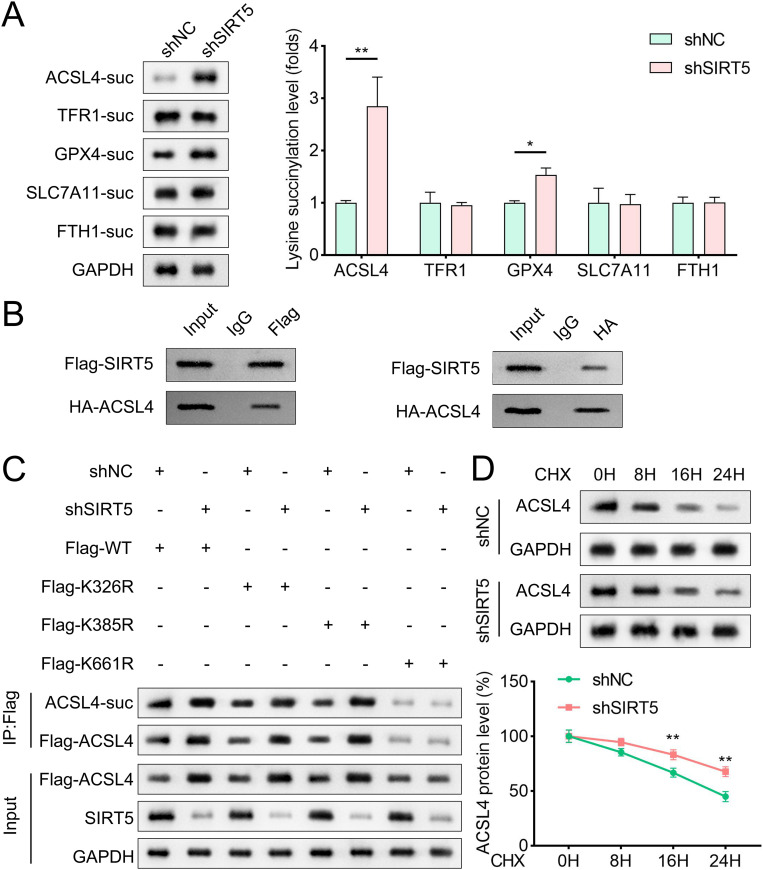
SIRT5 interacts with ACSL4 and regulates its succinylation status. (A) Western blot was carried out to evaluate the succinylation level of ferroptosis-related proteins in chondrocytes transfected with shNC or shSIRT5, n = 3. (B) CO-IP was performed to verify the endogenous binding relationship between SIRT5 and ACSL4. Cell lysates were incubated with SIRT5-specific antibodies and protein A/G agarose beads. The immunoprecipitated proteins were analyzed by Western blot using ACSL4-specific antibodies, n = 3. (C) The succinylation level of ACSL4 protein in chondrocytes (transfected with shNC or shSIRT5) after muation at the predicted succinylation sites, n = 3. (D) Quantitative analysis of protein degradation of ACSL4 when SIRT5 was inhibited. Protein levels of ACSL4 were determined at different time points after inhibiting SIRT5 expression, n = 3. Data were presented as mean ± SD. Comparisons between groups were performed using Student’s t-test. *p < 0.05, **p < 0.01.

**Fig 5:**
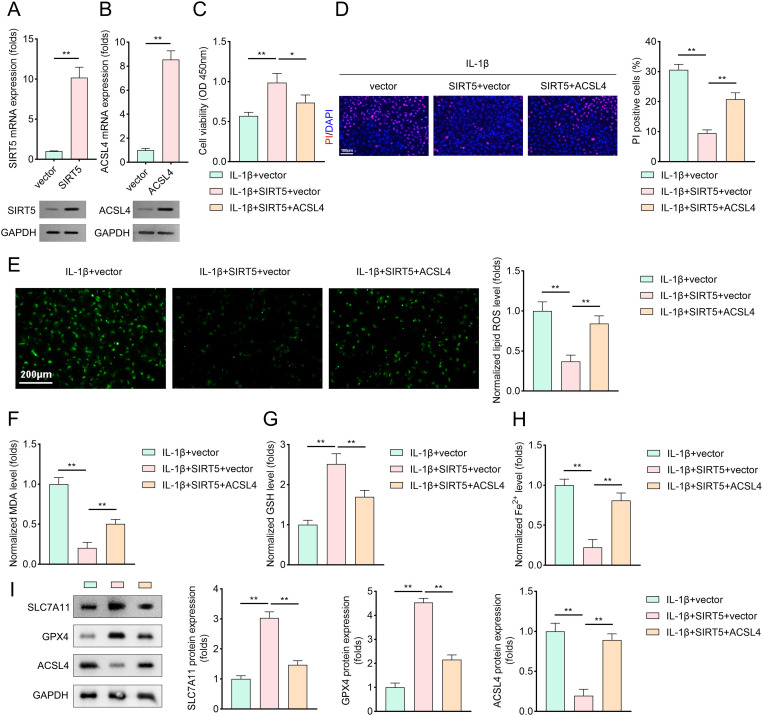
SIRT5-mediated cell ferroptosis is reversed by the elevation of ACSL4. (A–B) PCR and western blotting analysis were performed to evaluate the levels of SIRT5 and ACSL4 in chondrocytes transfected with vector or SIRT5. Total RNA and protein samples were collected, and SIRT5 and ACSL4 levels were determined using PCR and Western blotting, n = 3. (C) Cell viability of IL-1β treated chondrocytes (transfected with vector or SIRT5) obtained by CCK-8 method, n = 3. (D) DAPI/PI staining method was applied to evaluate cell death of IL-1β treated chondrocytes (transfected with vector or SIRT5), n = 3. (red: PI-labeled dead cells; blue: DAPI-labeled nuclei). (E–H) Cell ferroptosis of IL-1β treated chondrocytes (transfected with vector or SIRT5) was accessed by evaluating ROS, MDA, GSH, and Fe^2+^ levels, n = 3. (I) Representative protein bands and quantitative analysis of GPX4 and ACSL4 levels in IL-1β treated chondrocytes (transfected with vector or SIRT5), n = 3. Data were presented as mean ± SD. Comparisons between groups were performed using Student’s t-test or one-way ANOVA followed by Tukey’s post-hoc test. *p < 0.05, **p < 0.01.

To elucidate the mechanism underlying SIRT5-mediated protection against ferroptosis, we examined the succinylation status of ferroptosis-related proteins under SIRT5 knockdown. Results demonstrated that SIRT5 inhibition significantly increased ACSL4 lysine succinylation levels (Fig 4A). Furthermore, overexpression of SIRT5, however, decreased the ACSL4 lysine succinylation levels (S1 Fig). CO-IP assays further confirmed a direct interaction between SIRT5 and ACSL4 (Fig 4B). Bioinformatic analysis via the GPSuc server (http://kurata14.bio.kyutech.ac.jp/GPSuc/index.php) predicted three potential succinylation sites on ACSL4: K326, K385, and K661. Site-directed mutagenesis experiments revealed that SIRT5 inhibition increased ACSL4 succinylation in wild-type (WT), K326A, and K385A mutant groups, while the K661A mutation abolished this effect (Fig 4C). Additionally, SIRT5 inhibition stabilized ACSL4 protein by suppressing its degradation (Fig 4D). To further validate the role of SIRT5 and ACSL4, we overexpressed SIRT5 and ACSL4 in chondrocytes. Western blot analysis confirmed successful transfection (Fig 5A–5B). Overexpression of SIRT5 significantly enhanced chondrocyte viability and reduced PI-stained cell counts (Fig 5C–5D). However, ACSL4 overexpression abrogated these protective effects. Ferroptosis-related parameters were further analyzed: SIRT5 overexpression reduced lipid ROS, MDA, Fe2+ , and ACSL4 levels while increasing SLC7A11 and GPX4 expression. Notably, ACSL4 overexpression reversed these beneficial effects of SIRT5 overexpression (Fig 5E–5I).

### Cur alleviate OA progress *in vivo*

**Fig 6:**
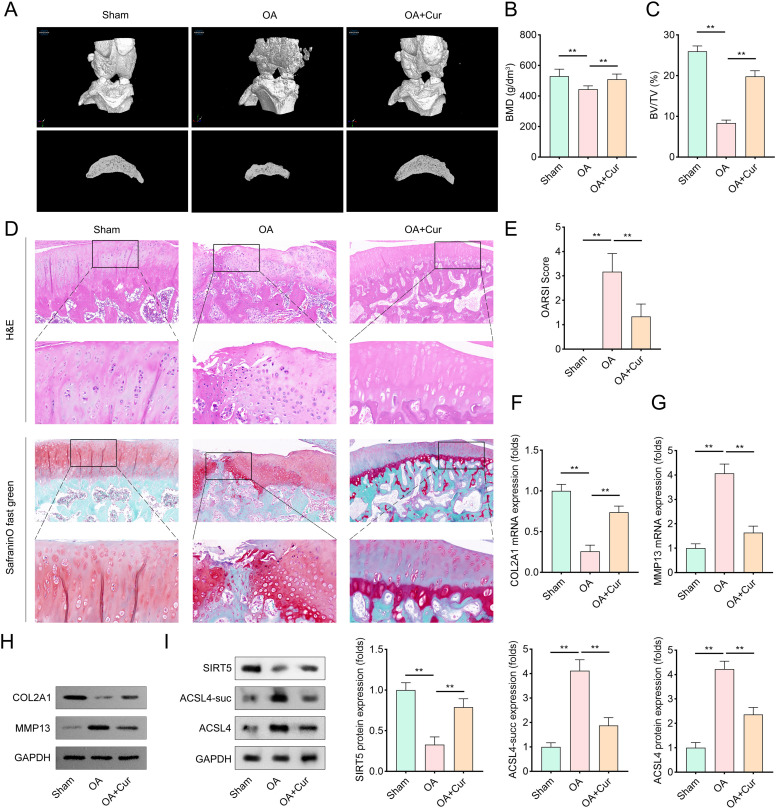
Cur alleviate OA progress *in vivo.* (A) Representative CT images of the femurs of rats. Micro-CT scanning was performed to visualize the bone structure of rat femurs. (B–C) Quantitative analysis of bone mineral density (BMD), and bone volume to tissue volume (BV/TV) were measured. BMD and BV/TV values were calculated from Micro-CT images, n = 6. (D) Representative images of H&E staining and Saffron O-solid green staining (red indicates cartilage tissues, and green indicates mineralized bone tissue sites). (E) The OARSI score assessed the degree of articular cartilage injury and the progression of OA. OARSI scoring was performed by blinded observers using a standardized scoring system, n = 6. (F–G) PCR analysis was performed to evaluate the mRNA levels of COL2A1 and MMP13 in cartilage tissues, n = 6. (H) Western blot analysis was performed to evaluate the protein levels of COL2A1 and MMP13 in cartilage tissues, n = 6. (I) Representative protein bands and quantitative analysis of SIRT5 and ACSL4 levels in cartilage tissues, n = 6. Data were presented as mean ± SD. Comparisons between groups were performed using one-way ANOVA followed by Tukey’s post-hoc test. **p < 0.01.

To evaluate Cur’s therapeutic effects in an OA model, we conducted in vivo experiments using OA-induced rats. Micro-CT analysis revealed significant cartilage destruction, roughened bone surfaces, and bone erosion in OA rats (Fig 6A–6C). In contrast, Cur treatment notably mitigated these structural abnormalities, preserving cartilage integrity and bone architecture. Histological evaluation via H&E staining and safranin O/fast green staining further demonstrated that Cur treatment alleviated OA-related cartilage damage and restored cartilage thickness (Fig 6D–6E). At the molecular level, COL2A1, a key cartilage matrix protein, was downregulated in OA rats but significantly upregulated by Cur treatment (Fig 6F,6H). Conversely, MMP13, a matrix-degrading enzyme implicated in OA progression, was highly expressed in OA rats but suppressed by Cur administration (Fig 6G,6H). Additionally, immunohistochemistry revealed that SIRT5 protein levels were reduced, while ACSL4 succinylation was elevated in OA cartilage tissues. Cur treatment reversed these changes: it upregulated SIRT5 expression and reduced ACSL4 succinylation (Fig 6I). Collectively, these findings indicate that Cur exerts protective effects against OA progression by modulating SIRT5-ACSL4 signaling.

## Discussion

In this study, we demonstrated that Cur treatment enhances chondrocyte viability and suppresses ferroptosis *in vitro*, while mitigating OA progression by reducing cartilage and bone destruction *in vivo*. Intriguingly, Cur exhibits strong structural correlation with SIRT5 protein. Inhibition of SIRT5 significantly attenuated the protective effects of Cur on chondrocytes. Furthermore, SIRT5 reduced ACSL4 succinylation, and ACSL4 overexpression potently reversed the anti-ferroptotic effects of SIRT5 upregulation in chondrocytes.

Cur has gained widespread application in both basic research and clinical management of OA owing to its multifaceted biological activities, including potent antioxidant, anti-inflammatory properties, and inhibition of MMP secretion, coupled with its broad pharmacological effects [30]. Ferroptosis, an iron-dependent form of regulated cell death characterized by excessive lipid ROS accumulation and mitochondrial morphological alterations, has been increasingly recognized as a critical driver of OA pathogenesis [10,31]. Experimental evidence demonstrates that ferroptosis is actively involved in OA progression, as observed in murine OA models, where it promotes upregulation of MMP-13 and downregulation of collagen type II alpha 1 chain (COL2A1) in chondrocytes [32]. Notably, intra-articular administration of Ferrostatin-1, a ferroptosis inhibitor, effectively suppresses ROS accumulation and mitigates OA progression [33]. However, the precise molecular mechanisms underlying chondrocyte ferroptosis in OA cartilage degeneration remain poorly defined. In this study, Cur was found to inhibit IL-1β-induced ferroptosis in chondrocytes, so it is important to find the key molecules of Cur regulating ferroptosis in chondrocytes.

Succinylation is a newly discovered post-translational modification involving the covalent addition of a succinyl group to lysine residues, which alters protein structure and function [34,35]. Dysregulation of succinylation in diverse proteins can disrupt multiple metabolic pathways, thereby promoting the development of inflammatory diseases [36]. For instance, succinylation of the TP53-induced glycolysis and apoptosis regulator (TIGAR) inhibits succinate dehydrogenase activity, leading to reduced ROS production, decreased MitoROS levels, and alleviation of lipid peroxidation in neurons—mechanisms that mitigate ferroptosis [22]. These findings underscore the critical role of succinylation in modulating ferroptosis, suggesting that targeting succinylation of ferroptosis-related proteins could be a therapeutic strategy for diseases such as OA. Sirtuins, a family of evolutionarily conserved deacylases (SIRT1–7), regulate diverse biological processes through deacetylation and desuccinylation of target proteins [37,38]. Among them, SIRT5 stands out as a dual-function enzyme capable of removing acetyl and succinyl groups from proteins, thereby modulating mitochondrial metabolism and redox homeostasis [39,40]. Notably, SIRT5 has been implicated in ferroptosis regulation, particularly in ischemia-reperfusion injury, where its desuccinylase activity safeguards mitochondrial function and suppresses lipid peroxidation [41]. Based on these findings, we propose that SIRT5 and its desuccinylase activity play a critical regulatory role in chondrocyte ferroptosis. Our experimental data demonstrate that SIRT5 expression is markedly downregulated in OA model cells, while Cur treatment effectively restores both SIRT5 expression and enzymatic activity.

ACSL4, a key lipid-metabolizing enzyme that promotes ferroptosis, represents an important pharmacological target for treating ferroptosis-related diseases. Previous studies have demonstrated that modulation of ACSL4 can inhibit chondrocyte ferroptosis in OA [42]. Building on this evidence, we hypothesized that ACSL4 activity may be regulated through succinylation modifications, potentially influencing chondrocyte ferroptosis. Our findings reveal that ACSL4 physically interacts with SIRT5, a known desuccinylase, and that SIRT5-mediated desuccinylation of ACSL4 protects chondrocytes from IL-1β-induced damage. These results suggest that targeting the ACSL4/SIRT5 axis may offer a novel therapeutic strategy for OA prevention and treatment.

## Conclusion

In summary, our findings demonstrate that Cur attenuates OA progression by enhancing SIRT5-mediated desuccinylation of ACSL4, thereby inhibiting chondrocyte ferroptosis. This study identifies Cur as a promising therapeutic compound that targets the ACSL4/SIRT5 axis for OA intervention.

## Supporting information

S1 Fig The ACSL4 lysine succinylation levels in chondrocytes with overexpressed SIRT5. (TIF)

S1 File Original data. (DOCX)

S1 File Raw_images. (PDF)
