# Supplementary figures and images for: Curcumin inhibits ferroptosis through dessuccinylation of SIRT5-associated ACSL4 protein, and plays a chondroprotective role in osteoarthritis
Source: PLoS One. 2025 Aug 18;20(8):e0328139. doi: 10.1371/journal.pone.0328139 (PMC12360603; doi:10.1371/journal.pone.0328139)

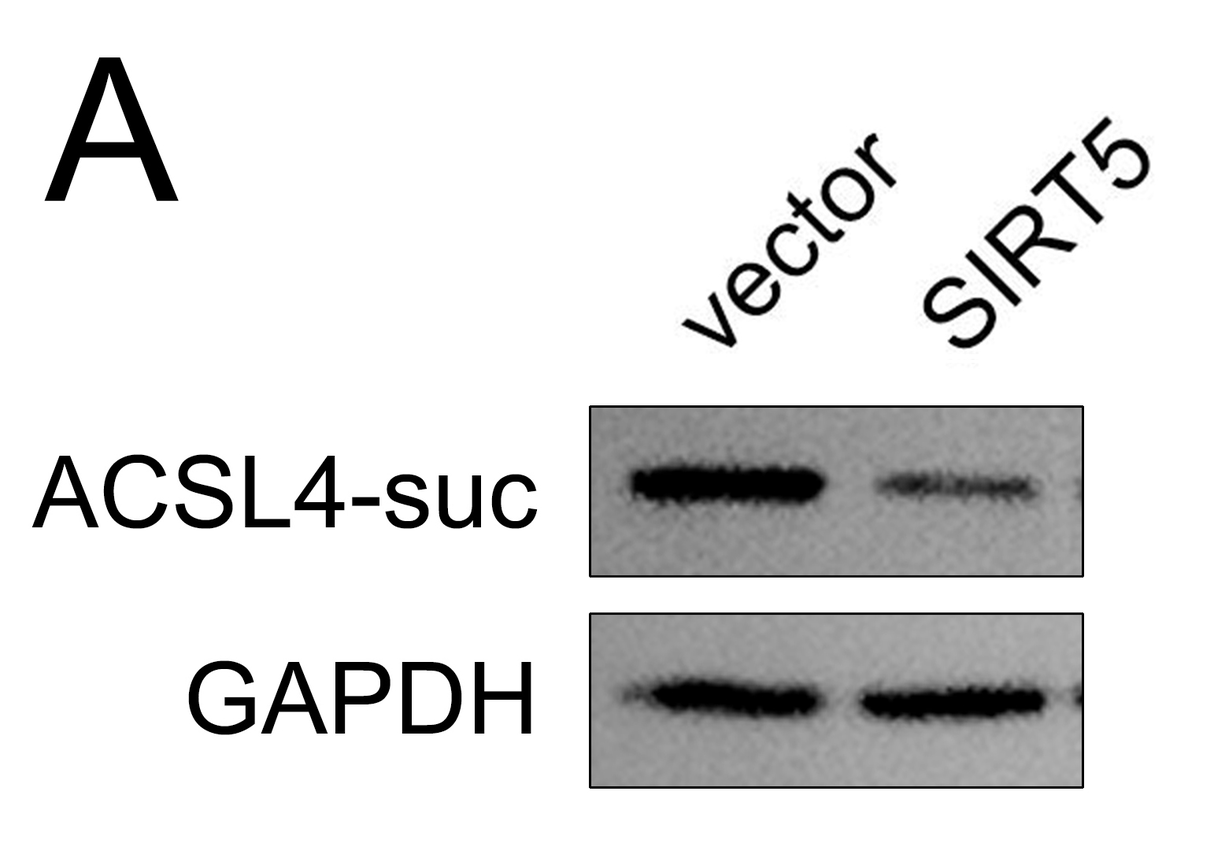

Supplement: S1 Fig — (TIF) [file pone.0328139.s001.tif]
