# Supplementary material for: Curcumin inhibits ferroptosis through dessuccinylation of SIRT5-associated ACSL4 protein, and plays a chondroprotective role in osteoarthritis
Source: PLoS One. 2025 Aug 18;20(8):e0328139. doi: 10.1371/journal.pone.0328139 (PMC12360603; doi:10.1371/journal.pone.0328139)
Supplement: S1 File — (DOCX) [file pone.0328139.s002.docx]

| Figure1C | Ctrl | IL-1β | IL-1β+PBS | IL-1β+Cur |
| --- | --- | --- | --- | --- |
| PI/DAPI | 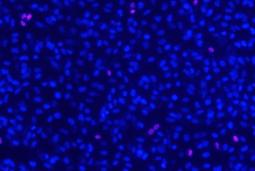 | 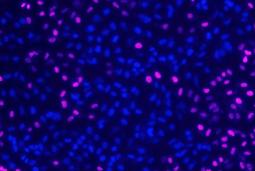 | 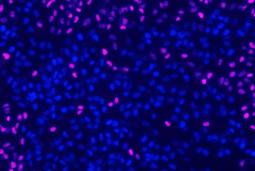 | 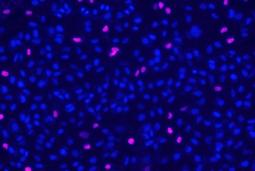 |

| Figure1E | Ctrl | IL-1β | IL-1β+PBS | IL-1β+Cur |
| --- | --- | --- | --- | --- |
|  | 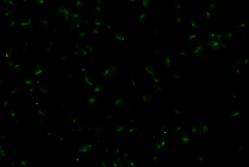 | 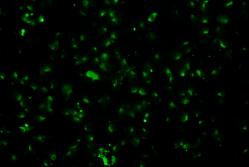 | 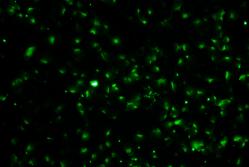 | 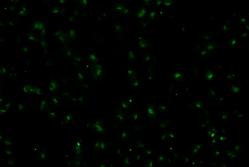 |

| Figure1I | Ctrl | IL-1β | IL-1β+PBS | IL-1β+Cur |
| --- | --- | --- | --- | --- |
| SLC7A11  55kDa | 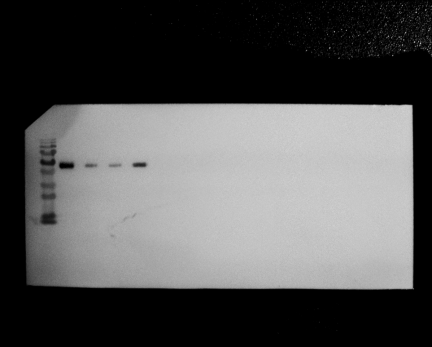 | | | |
| GPX4  17kDa | 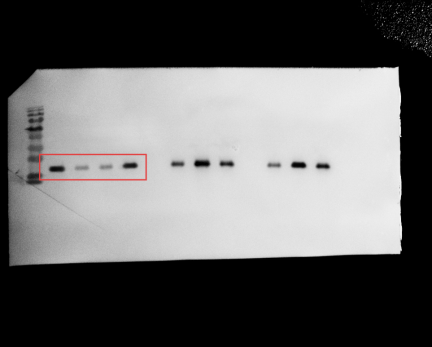 | | | |
| ACSL4  79kDa | 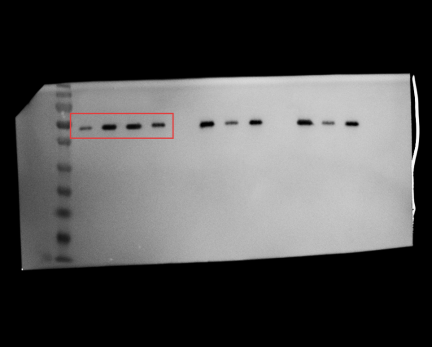 | | | |
| GAPDH  36kDa | 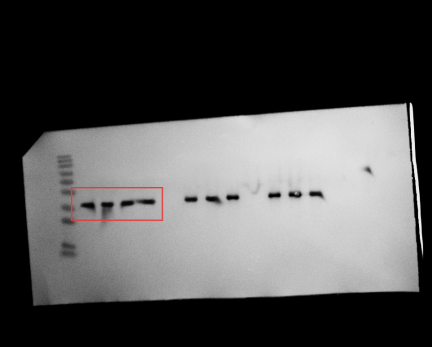 | | | |

| Figure2A | Ctrl | IL-1β | IL-1β+PBS | IL-1β+Cur |
| --- | --- | --- | --- | --- |
| Ksuc  10-180kDa | 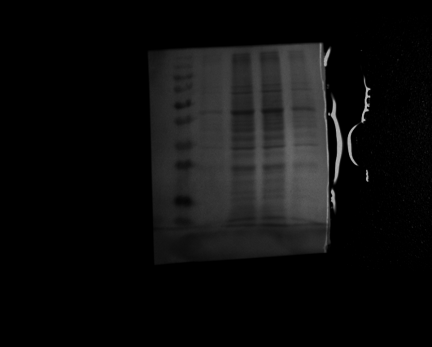 | | | |
| GAPDH  36kDa | 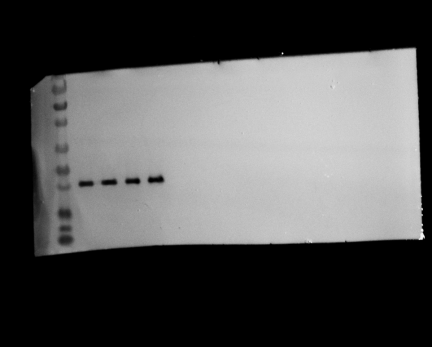 | | | |

| Figure2B | Ctrl | IL-1β | IL-1β+PBS | IL-1β+Cur |
| --- | --- | --- | --- | --- |
| KAT2A  94kDa | 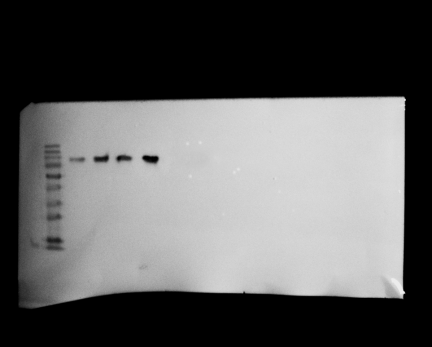 | | | |
| KAT3B  300kDa | 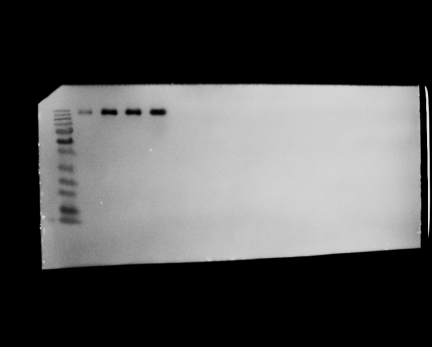 | | | |
| CPT1A  88kDa | 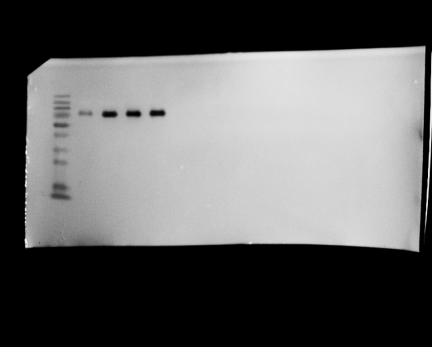 | | | |
| HAT1  50kDa | 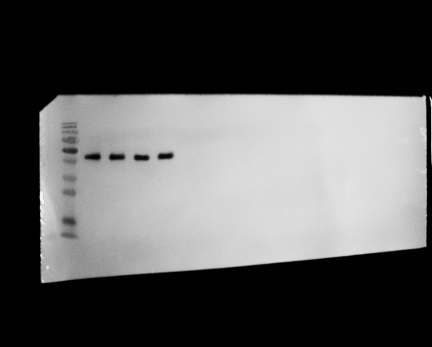 | | | |
| SIRT5  30kDa | 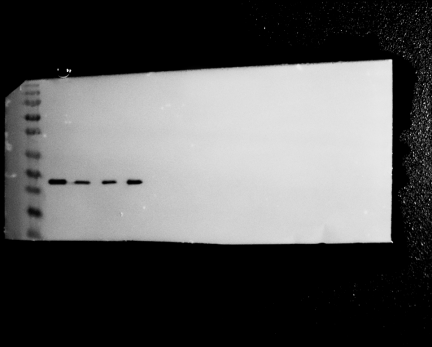 | | | |
| SIRT7  45kDa | 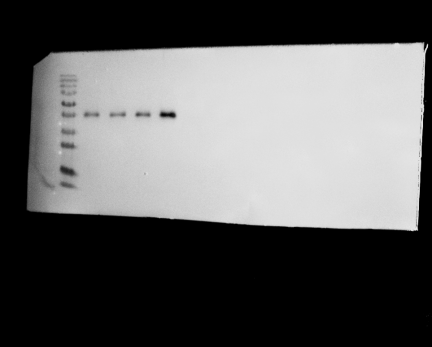 | | | |
| GAPDH  36kDa | 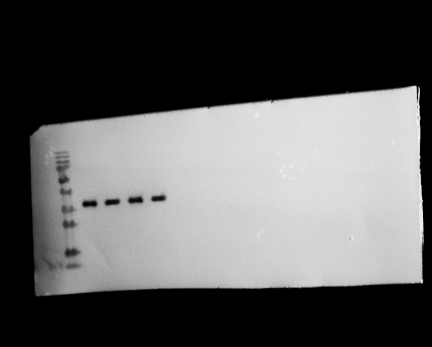 | | | |

| Figure3A | shNC | shSIRT5 |
| --- | --- | --- |
| SIRT5  30kDa | 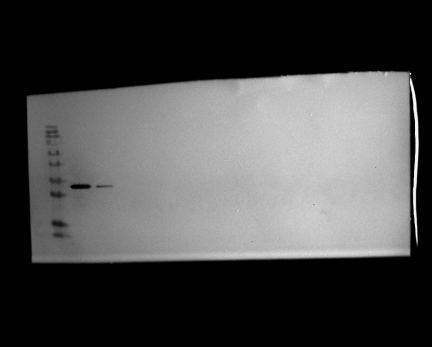 | |
| GAPDH  36kDa | 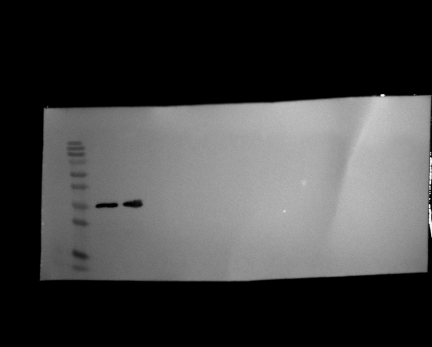 | |

| Figure3C | IL-1β+PBS+shNC | IL-1β+Cur+shNC | IL-1β+Cur+shSIRT5 |
| --- | --- | --- | --- |
| PI/DAPI | 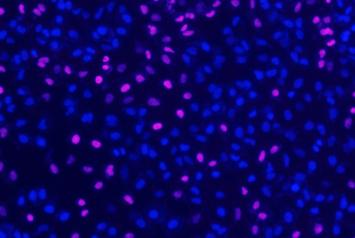 | 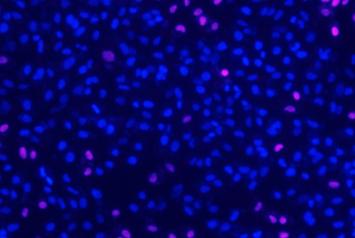 | 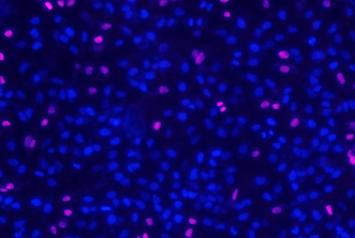 |

| Figure3D | IL-1β+PBS+shNC | IL-1β+Cur+shNC | IL-1β+Cur+shSIRT5 |
| --- | --- | --- | --- |
|  | 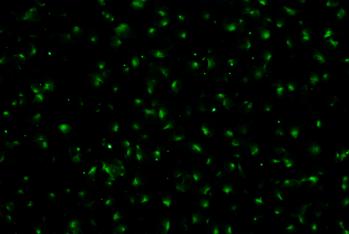 | 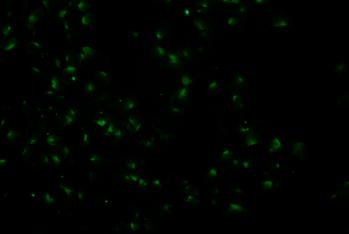 | 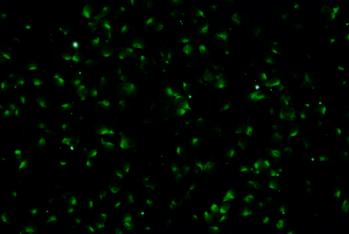 |

| Figure3H | IL-1β+PBS+shNC | IL-1β+Cur+shNC | IL-1β+Cur+shSIRT5 |
| --- | --- | --- | --- |
| SLC7A11  55kDa | 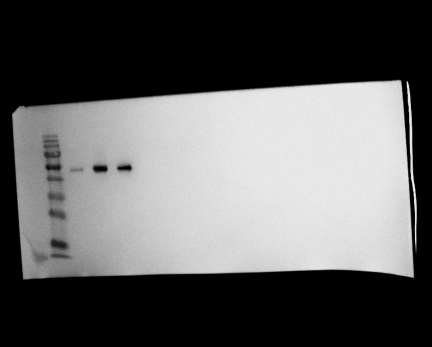 | | |
| GPX4  17kDa | 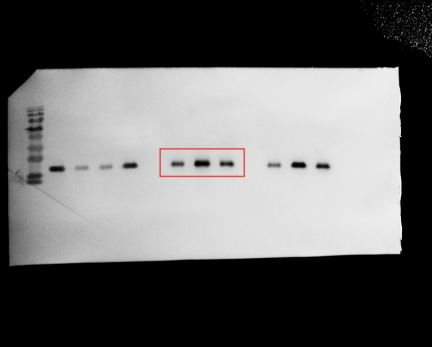 | | |
| ACSL4  79kDa | 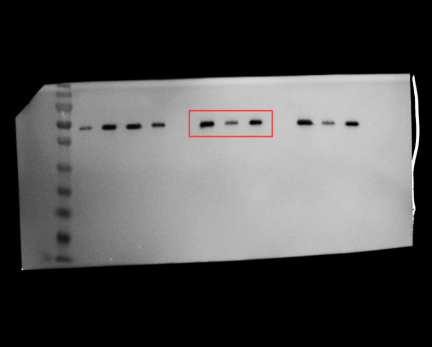 | | |
| GAPDH  36kDa | 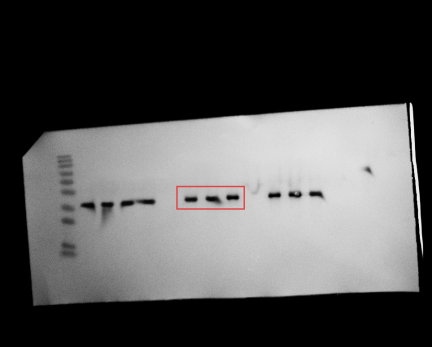 | | |

| Figure4A | shNC | shSIRT5 |
| --- | --- | --- |
| ACSL4-suc  79kDa | 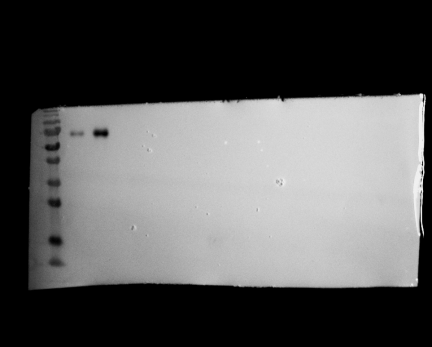 | |
| TFR1--suc  90kDa | 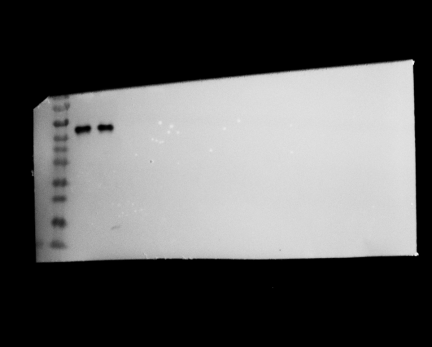 | |
| GPX4-suc  17kDa | 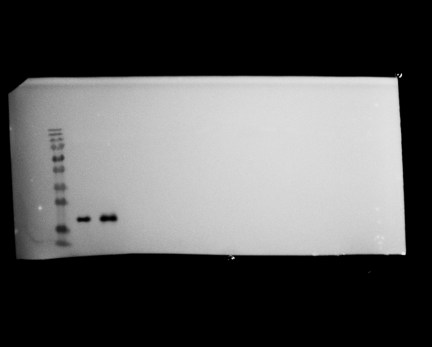 | |
| SLC7A11-suc  55kDa | 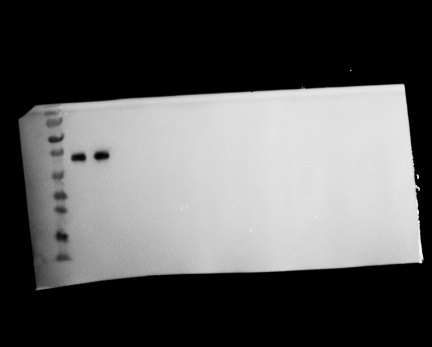 | |
| FTH1-suc  21kDa | 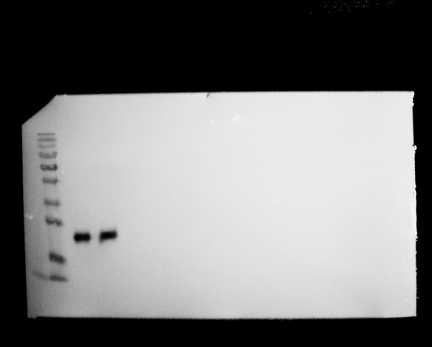 | |
| GAPDH  36kDa | 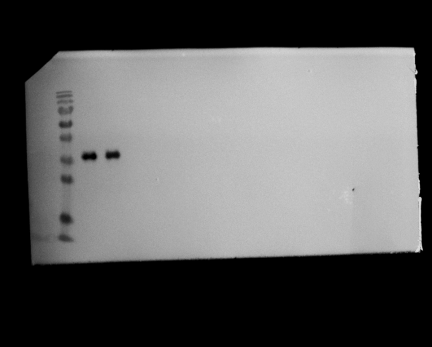 | |

| Figure4B | Input | IgG | Flag | Input | IgG | HA |
| --- | --- | --- | --- | --- | --- | --- |
| Flag-SIRT5  30kDa | 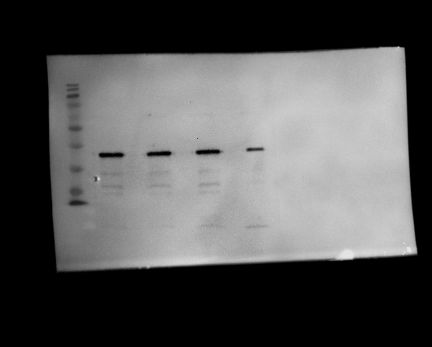 | | | | | |
| HA-ACSL4  79kDa | 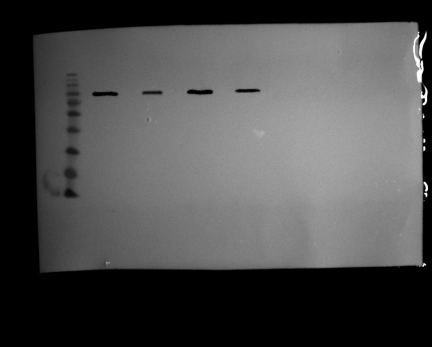 | | | | | |

| Figure4C | shNC | + | - | + | - | + | - | + | - |
| --- | --- | --- | --- | --- | --- | --- | --- | --- | --- |
|  | shSIRT5 | - | + | - | + | - | + | - | + |
|  | Flag-WT | + | + | - | - | - | - | - | - |
|  | Flag-K326R | - | - | + | + | - | - | - | - |
|  | Flag-K385R | - | - | - | - | + | + | - | - |
|  | Flag-K661R | - | - | - | - | - | - | + | + |
| IP:Flag | ACSL4-suc  79kDa | 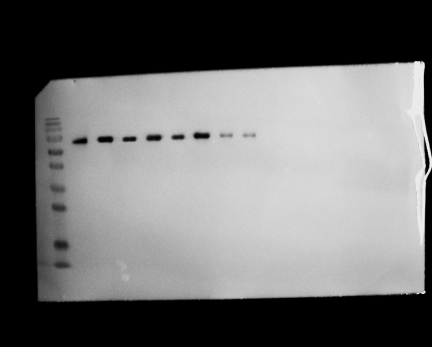 | | | | | | | |
|  | Flag-ACSL4  79kDa | 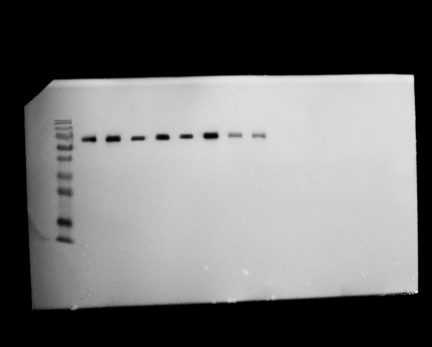 | | | | | | | |
| Input | Flag-ACSL4  79kDa | 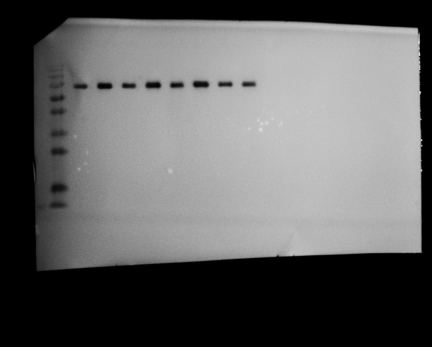 | | | | | | | |
|  | SIRT5  30kDa | 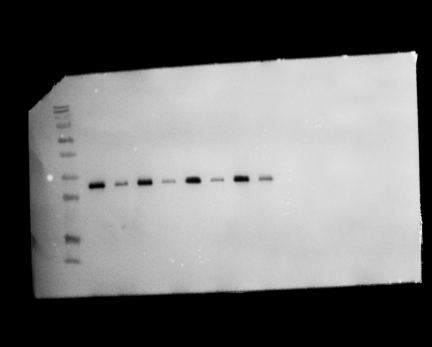 | | | | | | | |
|  | GAPDH  36kDa | 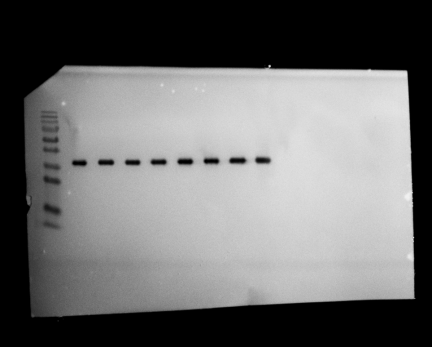 | | | | | | | |

| Figure4D | | 0H | 8H | 16H | 24H |
| --- | --- | --- | --- | --- | --- |
| CHX | |  |  |  |  |
| shNC | ACSL4  79kDa | 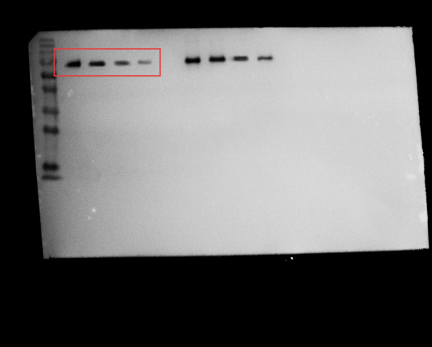 | | | |
|  | GAPDH  36kDa | 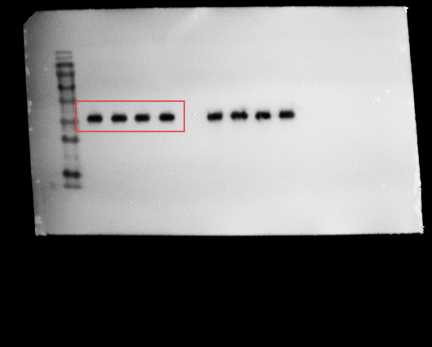 | | | |
| shSIRT5 | ACSL4  79kDa | 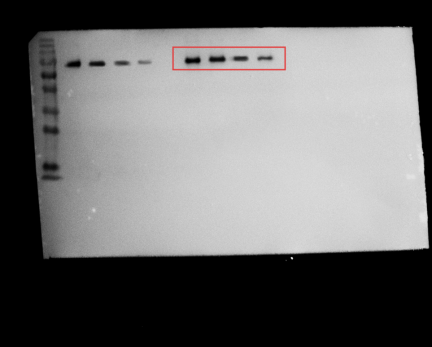 | | | |
|  | GAPDH  36kDa | 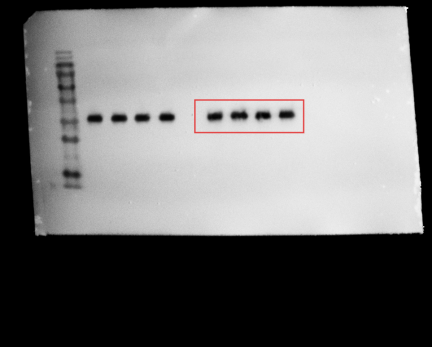 | | | |

| Figure5A | vector | SIRT5 |
| --- | --- | --- |
| SIRT5  30kDa | 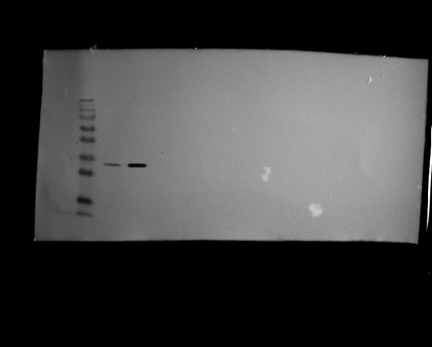 | |
| GAPDH  36kDa | 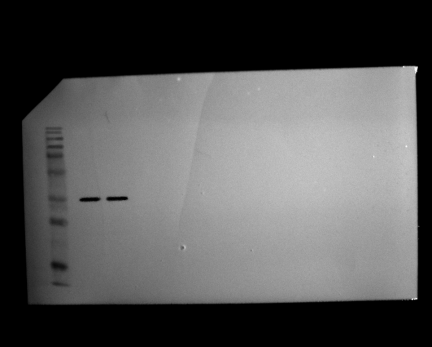 | |

| Figure5B | vector | ACSL4 |
| --- | --- | --- |
| ACSL4  79kDa | 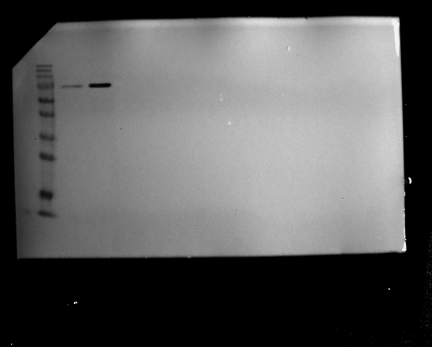 | |
| GAPDH  36kDa | 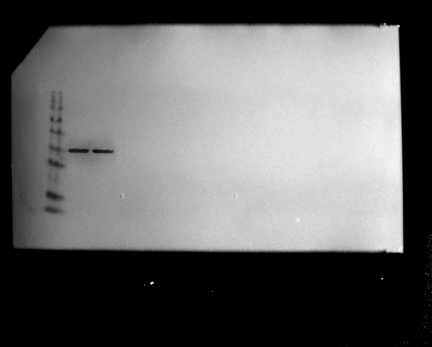 | |

| Figure5D | IL-1β | | |
| --- | --- | --- | --- |
|  | vector | SIRT5+vector | SIRT5+ACSL4 |
| PI/DAPI | 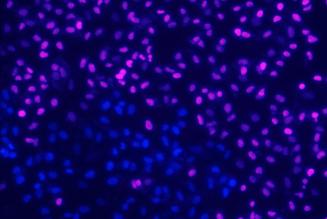 | 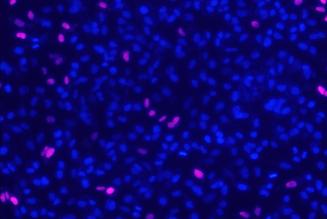 | 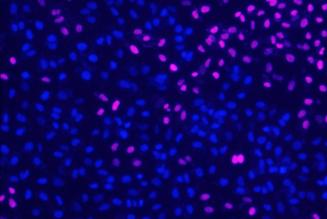 |

| Figure5E | IL-1β+vector | IL-1β+SIRT5+vector | IL-1β+SIRT5+ACSL4 |
| --- | --- | --- | --- |
|  | 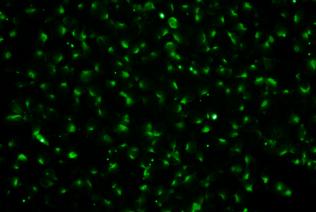 | 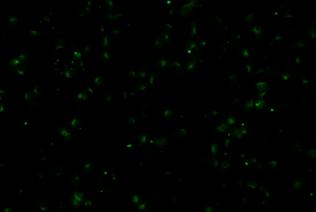 | 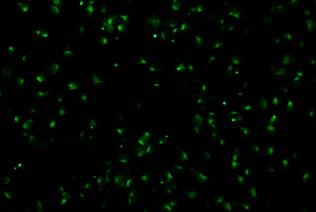 |

| Figure5I | IL-1β+vector | IL-1β+SIRT5+vector | IL-1β+SIRT5+ACSL4 |
| --- | --- | --- | --- |
| SLC7A11  55kDa | 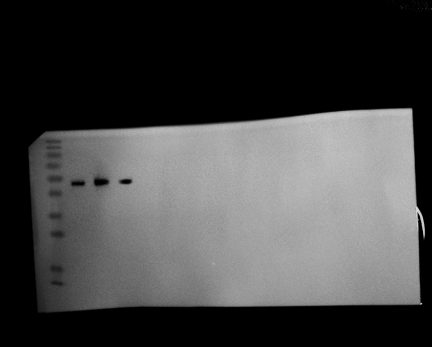 | | |
| GPX4  17kDa | 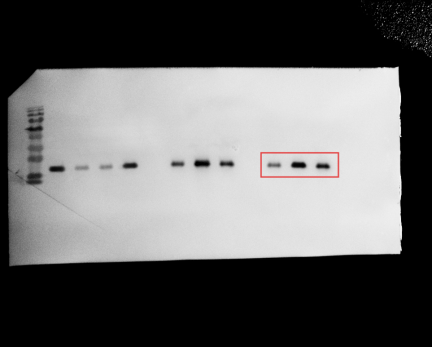 | | |
| ACSL4  79kDa | 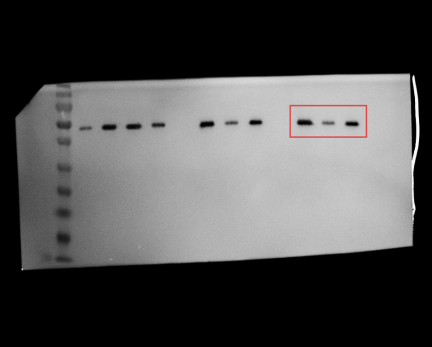 | | |
| GAPDH  36kDa | 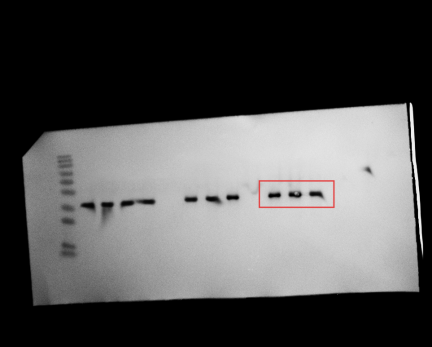 | | |

| Figure6A | Sham | OA | OA+Cur |
| --- | --- | --- | --- |
|  | 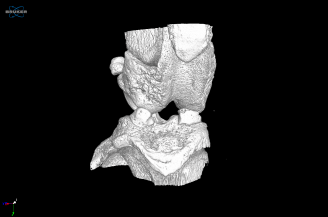 | 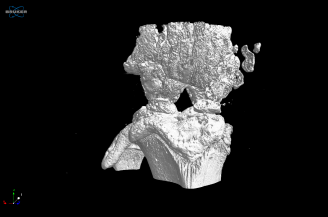 | 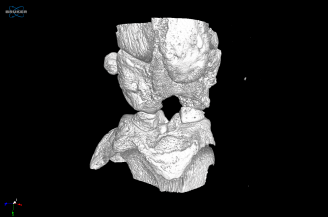 |
|  | 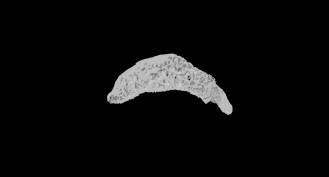 | 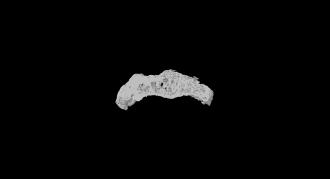 | 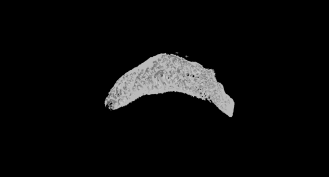 |

| Figure6D | Sham | OA | OA+Cur |
| --- | --- | --- | --- |
| H＆E | 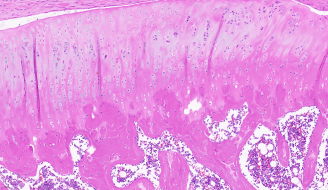 | 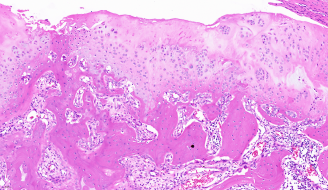 | 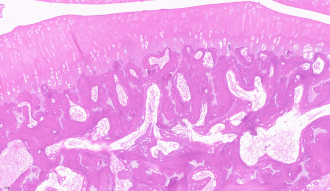 |
|  | 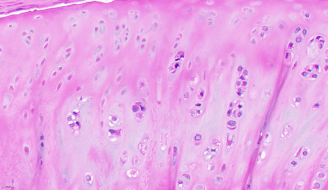 | 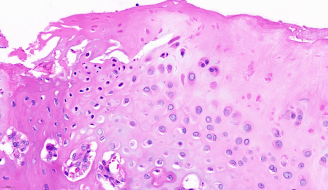 | 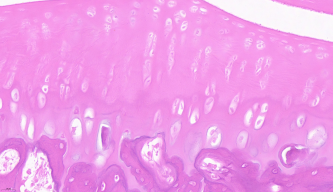 |
| SafraninO fast green | 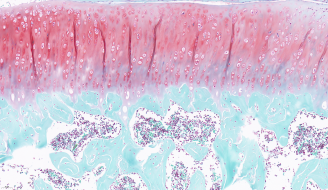 | 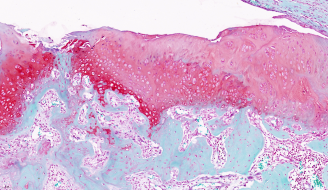 | 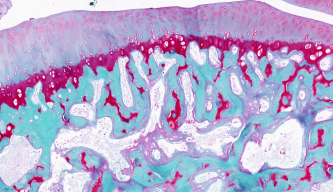 |
|  | 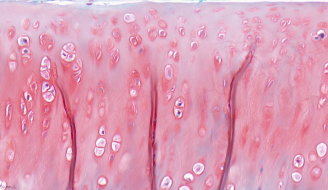 | 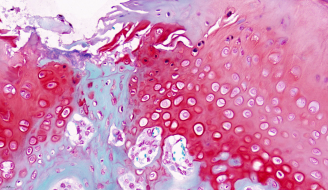 | 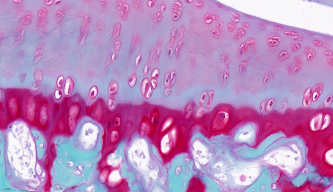 |

| Figure6H | Sham | OA | OA+Cur |
| --- | --- | --- | --- |
| COL2A1  142kDa | 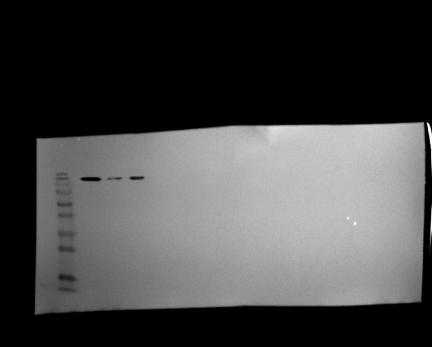 | | |
| MMP13  60kDa | 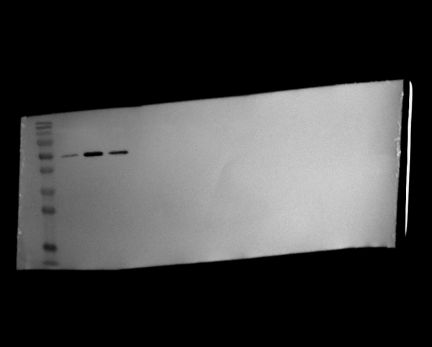 | | |
| GAPDH  36kDa | 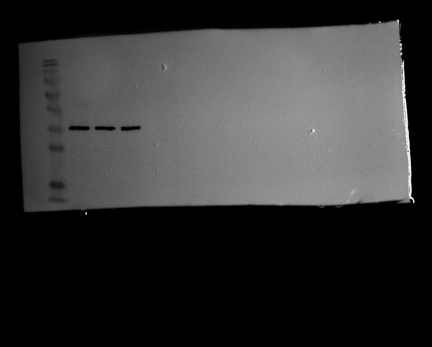 | | |

| Figure6I | Sham | OA | OA+Cur |
| --- | --- | --- | --- |
| SIRT5  30kDa | 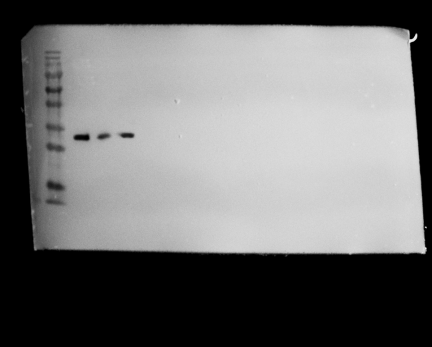 | | |
| ACSL4-suc  79kDa | 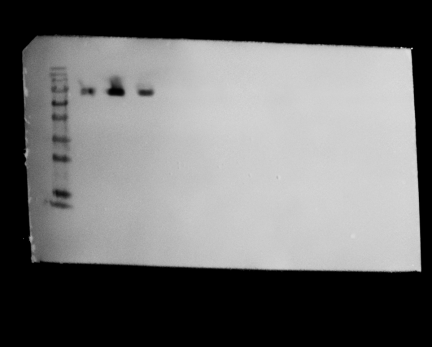 | | |
| ACSL4  79kDa | 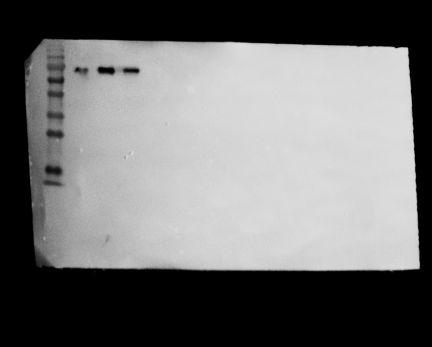 | | |
| GAPDH  36kDa | 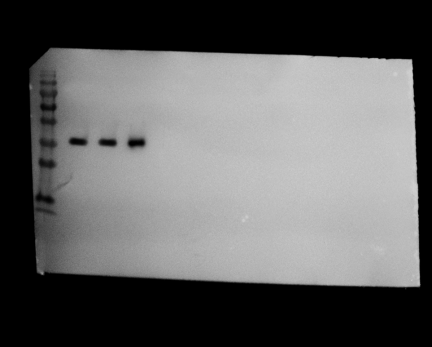 | | |

| Figure S1A | vector | SIRT5 |
| --- | --- | --- |
| ACSL4-suc  79kDa | 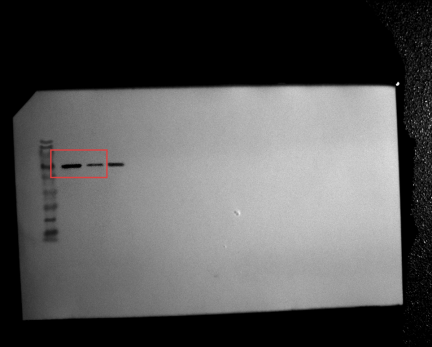 | |
| GAPDH  36kDa | 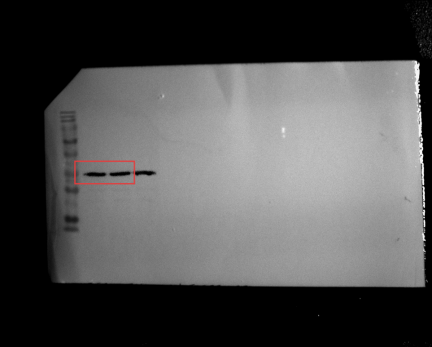 | |
