# Supplementary material for: Curcumin inhibits ferroptosis through dessuccinylation of SIRT5-associated ACSL4 protein, and plays a chondroprotective role in osteoarthritis
Source: PLoS One. 2025 Aug 18;20(8):e0328139. doi: 10.1371/journal.pone.0328139 (PMC12360603; doi:10.1371/journal.pone.0328139)
Supplement: S1 File — (PDF) [file pone.0328139.s003.pdf]

Figure1I	Ctrl	IL-1β	IL-1β+PBS	IL-1β+Cur
SLC7A11<br>55kDa	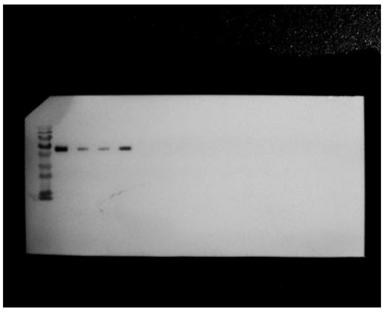			
GPX4<br>17kDa	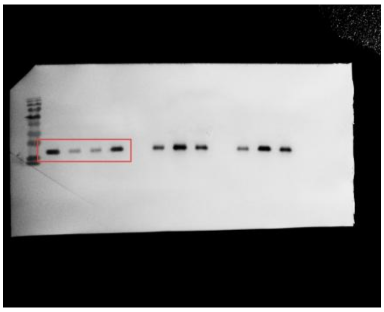			
ACSL4<br>79kDa	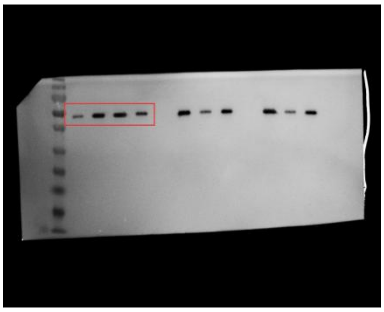			
GAPDH<br>36kDa	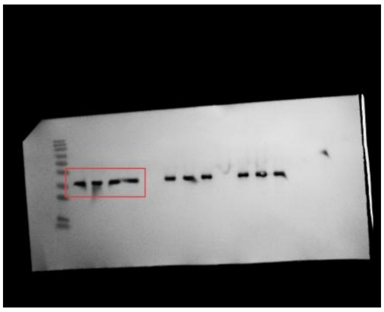			

Figure2A	Ctrl	IL-1β	IL-1β+PBS	IL-1β+Cur
Ksuc 10-180kDa	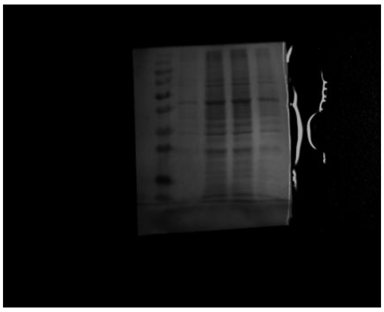			
GAPDH 36kDa	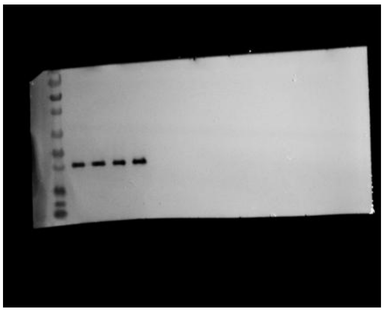			

Figure2B	Ctrl	IL-1β	IL-1β+PBS	IL-1β+Cur
KAT2A<br>94kDa	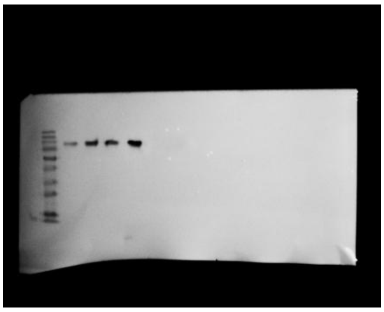			
KAT3B<br>300kDa	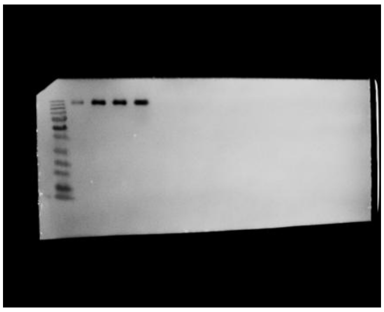			
CPT1A<br>88kDa	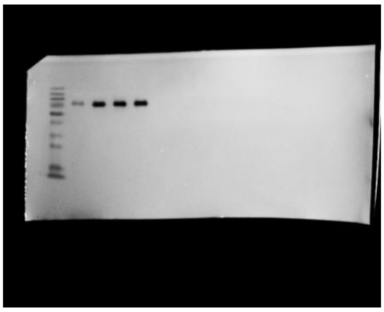			
HAT1<br>50kDa	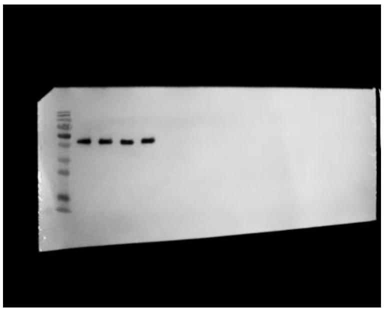			
SIRT5<br>30kDa	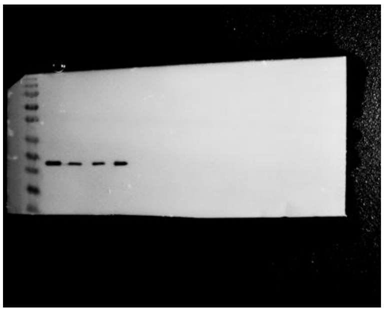			

SIRT7<br>45kDa	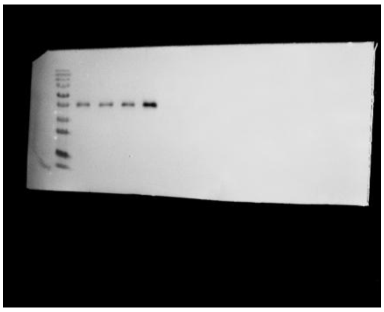
GAPDH<br>36kDa	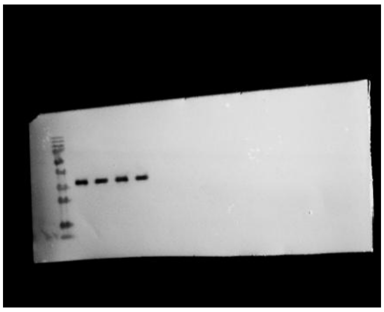

Figure3A	shNC	shSIRT5
SIRT5<br>30kDa	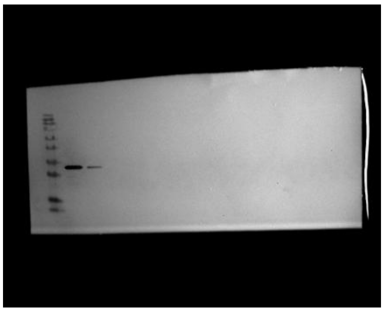	
GAPDH<br>36kDa	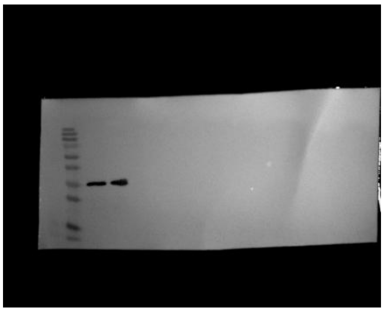	

Figure3H	IL-1β+PBS+shNC	IL-1β+Cur+shNC	IL-1β+Cur+shSIRT5
SLC7A11<br>55kDa	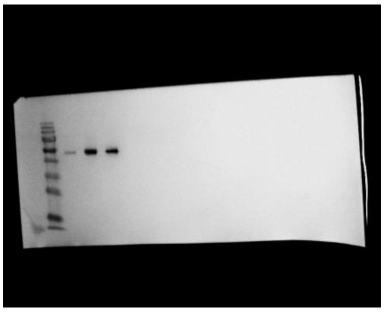		
GPX4<br>17kDa	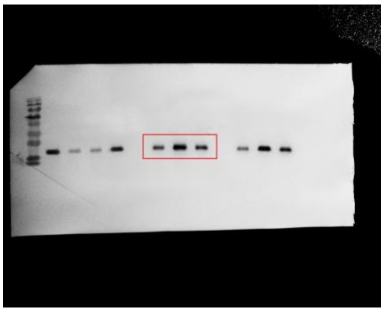		
ACSL4<br>79kDa	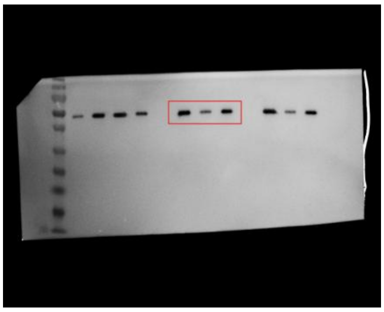		
GAPDH<br>36kDa	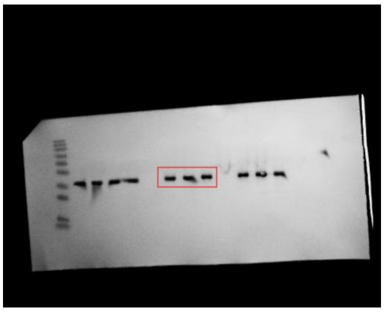		

Figure4A	shNC	shSIRT5
ACSL4-suc 79kDa	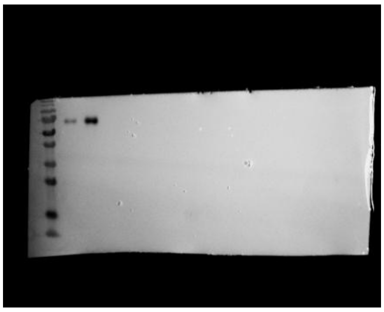	
TFR1--suc 90kDa	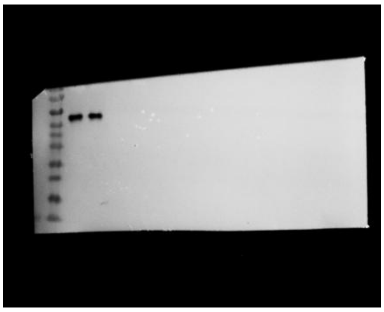	
GPX4-suc 17kDa	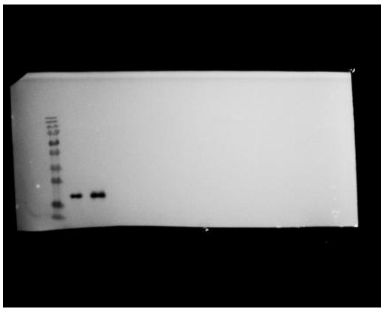	
SLC7A11-suc 55kDa	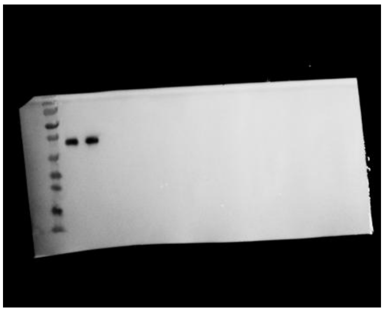	
FTH1-suc 21kDa	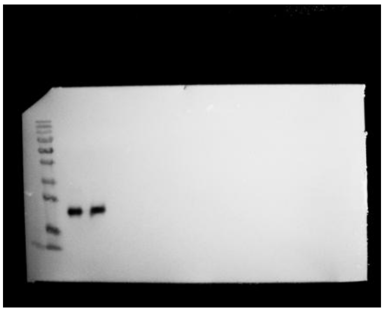	

GAPDH<br>36kDa	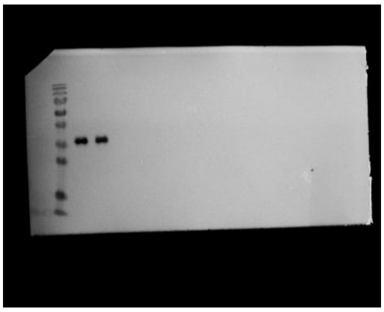

Figure4B	Input	IgG	Flag	Input	IgG	HA
Flag-SIRT5<br>30kDa	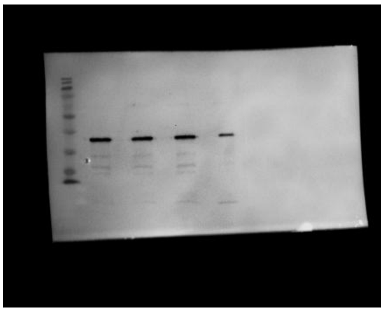					
HA-ACSL4<br>79kDa	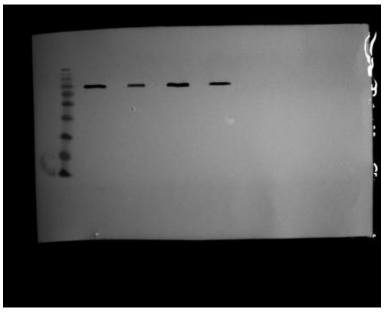					

<table>
<tr><td rowspan="6">Figure4C</td><td>shNC</td><td>+</td><td>-</td><td>+</td><td>-</td><td>+</td><td>-</td><td>+</td><td>-</td></tr>
<tr><td>shSIRT5</td><td>-</td><td>+</td><td>-</td><td>+</td><td>-</td><td>+</td><td>-</td><td>+</td></tr>
<tr><td>Flag-WT</td><td>+</td><td>+</td><td>-</td><td>-</td><td>-</td><td>-</td><td>-</td><td>-</td></tr>
<tr><td>Flag-K326R</td><td>-</td><td>-</td><td>+</td><td>+</td><td>-</td><td>-</td><td>-</td><td>-</td></tr>
<tr><td>Flag-K385R</td><td>-</td><td>-</td><td>-</td><td>-</td><td>+</td><td>+</td><td>-</td><td>-</td></tr>
<tr><td>Flag-K661R</td><td>-</td><td>-</td><td>-</td><td>-</td><td>-</td><td>-</td><td>+</td><td>+</td></tr>
<tr><td rowspan="2">IP:Flag</td><td>ACSL4-suc
79kDa</td><td colspan="8">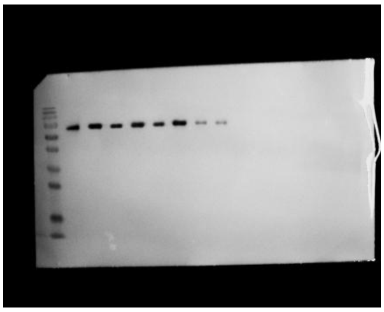</td></tr>
<tr><td>Flag-ACSL4
79kDa</td><td colspan="8">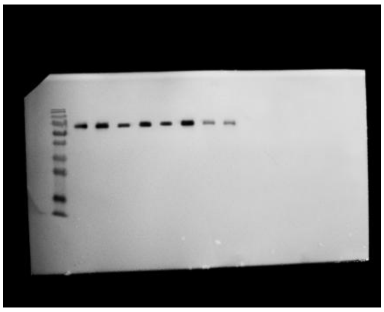</td></tr>
<tr><td rowspan="2">Input</td><td>Flag-ACSL4
79kDa</td><td colspan="8">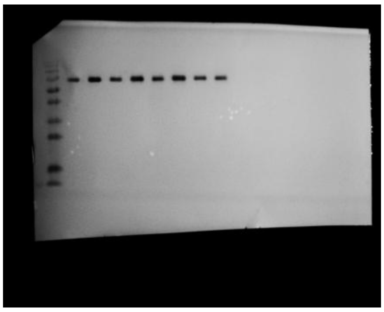</td></tr>
<tr><td>SIRT5
30kDa</td><td colspan="8">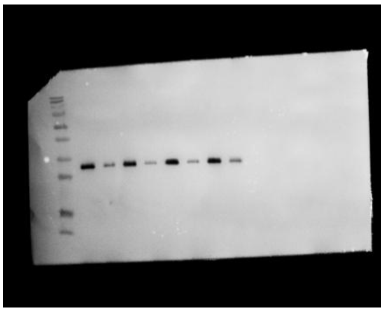</td></tr>
</table>

	GAPDH<br>36kDa	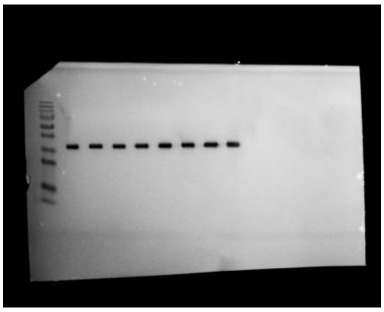

Figure4D		0H	8H	16H	24H
CHX					
shNC	ACSL4<br>79kDa	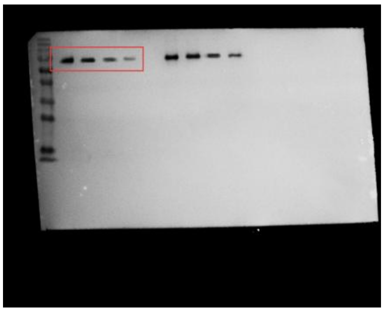			
	GAPDH<br>36kDa	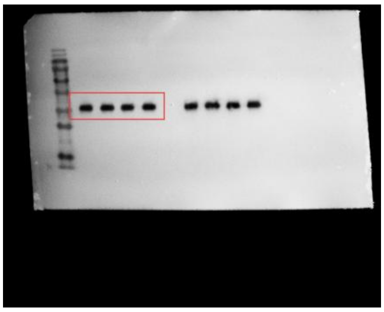			
shSIRT5	ACSL4<br>79kDa	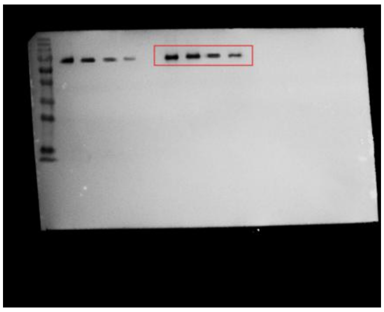			
	GAPDH<br>36kDa	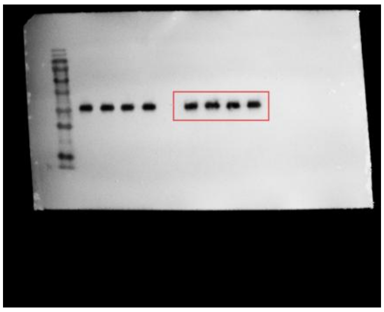			

<table>
<tr><td>Figure5A</td><td>vector</td><td>SIRT5</td></tr>
<tr><td>SIRT5
30kDa</td><td colspan="2">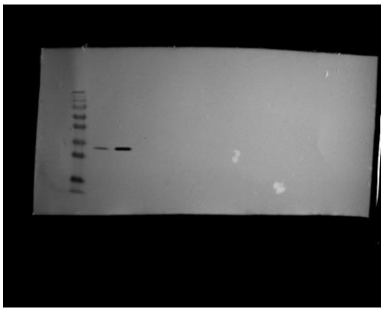</td></tr>
<tr><td>GAPDH
36kDa</td><td colspan="2">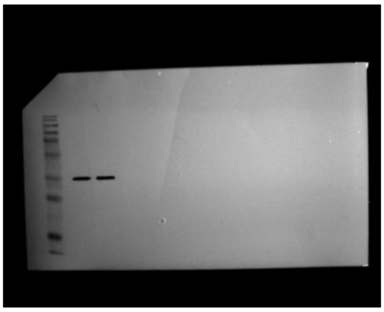</td></tr>
</table>

<table>
<tr><td>Figure5B</td><td>vector</td><td>ACSL4</td></tr>
<tr><td>ACSL4
79kDa</td><td colspan="2">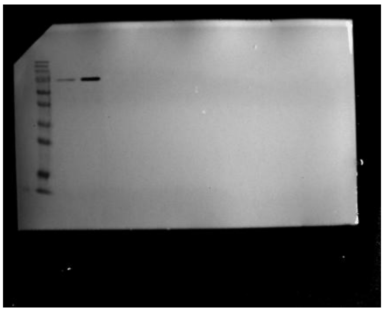</td></tr>
<tr><td>GAPDH
36kDa</td><td colspan="2">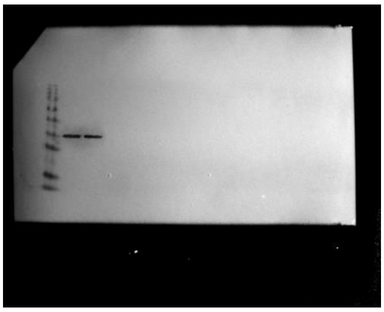</td></tr>
</table>

Figure5I	IL-1β+vector	IL-1β+SIRT5+vector	IL-1β+SIRT5+ACSL4
SLC7A11<br>55kDa	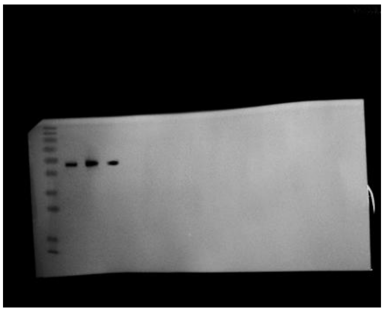		
GPX4<br>17kDa	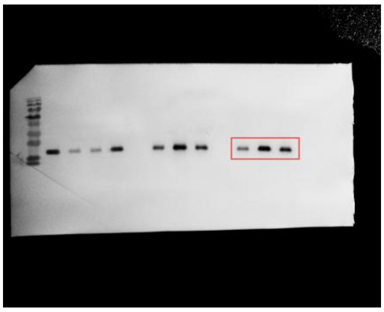		
ACSL4<br>79kDa	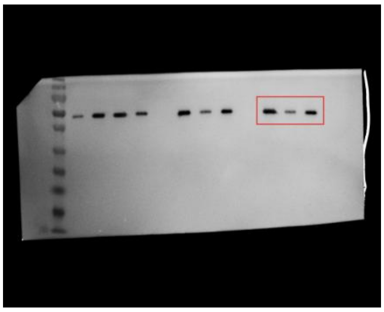		
GAPDH<br>36kDa	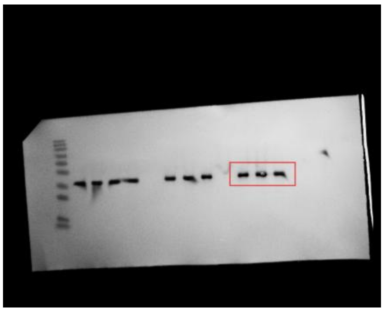		

Figure6H	Sham	OA	OA+Cur
COL2A1<br>142kDa	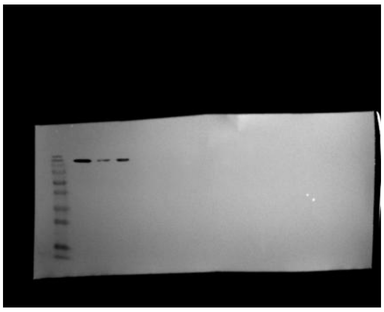		
MMP13<br>60kDa	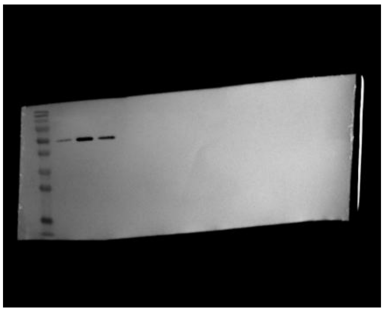		
GAPDH<br>36kDa	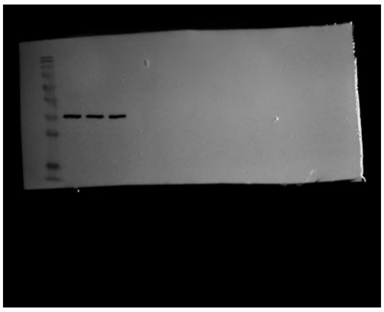		

Figure6I	Sham	OA	OA+Cur
SIRT5 30kDa	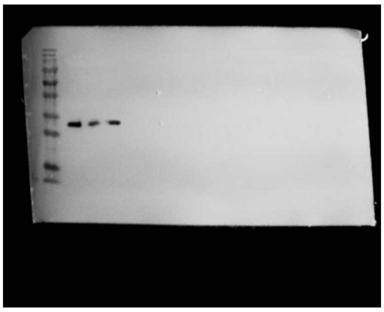		
ACSL4-suc 79kDa	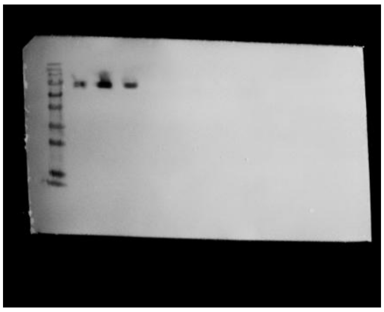		
ACSL4 79kDa	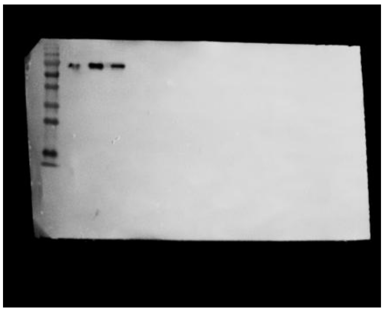		
GAPDH 36kDa	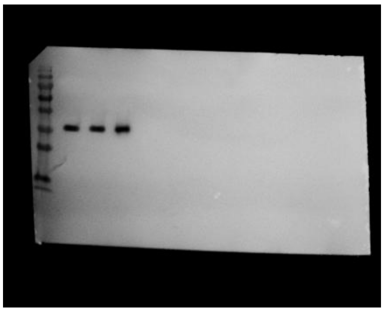		

Figure S1A	vector	SIRT5
ACSL4-suc 79kDa	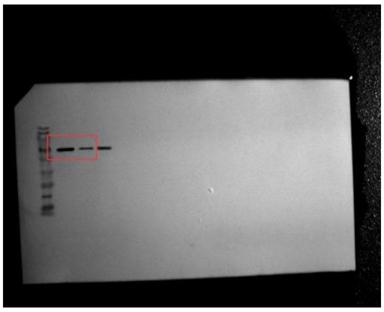	
GAPDH 36kDa	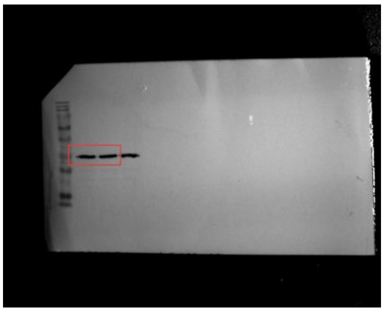	
